# Cyclic di-AMP regulation of osmotic homeostasis is essential in Group B Streptococcus

**DOI:** 10.1371/journal.pgen.1007342

**Published:** 2018-04-16

**Authors:** Laura Devaux, Dona Sleiman, Maria-Vittoria Mazzuoli, Myriam Gominet, Philippe Lanotte, Patrick Trieu-Cuot, Pierre-Alexandre Kaminski, Arnaud Firon

**Affiliations:** 1 Institut Pasteur, Unité Biologie des Bactéries Pathogènes à Gram-positif, CNRS ERL 6002, Paris, France; 2 Université Paris Diderot, Sorbonne Paris Cité, Paris, France; 3 Université de Tours, Infectiologie et Santé Publique, Bactéries et Risque Materno-Fœtal, INRA UMR1282, Tours France; 4 Hôpital Bretonneau, Centre Hospitalier Régional et Universitaire de Tours, Service de Bactériologie-Virologie, Tours France; Indiana University, UNITED STATES

## Abstract

Cyclic nucleotides are universally used as secondary messengers to control cellular physiology. Among these signalling molecules, cyclic di-adenosine monophosphate (c-di-AMP) is a specific bacterial second messenger recognized by host cells during infections and its synthesis is assumed to be necessary for bacterial growth by controlling a conserved and essential cellular function. In this study, we sought to identify the main c-di-AMP dependent pathway in *Streptococcus agalactiae*, the etiological agent of neonatal septicaemia and meningitis. By conditionally inactivating *dacA*, the only diadenyate cyclase gene, we confirm that c-di-AMP synthesis is essential in standard growth conditions. However, c-di-AMP synthesis becomes rapidly dispensable due to the accumulation of compensatory mutations. We identified several mutations restoring the viability of a Δ*dacA* mutant, in particular a loss-of-function mutation in the osmoprotectant transporter BusAB. Identification of c-di-AMP binding proteins revealed a conserved set of potassium and osmolyte transporters, as well as the BusR transcriptional factor. We showed that BusR negatively regulates *busAB* transcription by direct binding to the *busAB* promoter. Loss of BusR repression leads to a toxic *busAB* expression in absence of c-di-AMP if osmoprotectants, such as glycine betaine, are present in the medium. In contrast, deletion of the *gdpP* c-di-AMP phosphodiesterase leads to hyperosmotic susceptibility, a phenotype dependent on a functional BusR. Taken together, we demonstrate that c-di-AMP is essential for osmotic homeostasis and that the predominant mechanism is dependent on the c-di-AMP binding transcriptional factor BusR. The regulation of osmotic homeostasis is likely the conserved and essential function of c-di-AMP, but each species has evolved specific c-di-AMP mechanisms of osmoregulation to adapt to its environment.

## Author summary

Nucleotide-based second messengers play central functions in bacterial physiology and host-pathogen interactions. Among these signalling nucleotides, cyclic-di-AMP (c-di-AMP) synthesis was originally assumed to be essential for bacterial growth. In this study, we confirmed that the only di-adenylate cyclase enzyme in the opportunistic pathogen *Streptococcus agalactiae* is essential in standard growth conditions. However, c-di-AMP synthesis becomes rapidly dispensable by accumulating spontaneous mutations in genes involved in osmotic regulation. We identified that c-di-AMP binds directly to four proteins necessary to maintain osmotic homeostasis, including three osmolyte transporters and the BusR transcriptional factor. We demonstrated that BusR negatively controls the expression of the *busAB* operon and that it is the main component leading to growth inhibition in the absence of c-di-AMP synthesis if osmoprotectants are present in the environment. Overall, c-di-AMP is essential to maintain osmotic homeostasis by coordinating osmolyte uptake and thus bacteria have developed specific mechanisms to keep c-di-AMP as the central regulator of osmotic homeostasis.

## Introduction

Cyclic nucleotides are signalling molecules, commonly called second messengers, which regulate cellular processes by binding to targeted effectors [1–3]. Specific cyclic di-nucleotides are synthesized by prokaryotes and eukaryotes, and this specificity is exploited by host cells to monitor bacterial infections [4, 5]. For example, cyclic-di-AMP (c-di-AMP) is synthesized by almost all bacteria, except proteobacteria, and induces a type I interferon response through targeting the STING sensor. STING is also activated by the eukaryotic cyclic di-nucleotide 2’5’cGAMP that is generated in response to the presence of bacterial DNA in the host cytosol [6–9]. Some bacterial pathogens have evolved mechanisms to modulate the immune response to c-di-AMP [9, 10], but the understanding of the role of c-di-AMP in bacterial physiology and during infection remains limited.

Unlike other second messengers, the synthesis of c-di-AMP was originally assumed to be essential for bacterial growth in standard *in vitro* conditions [11, 12]. Yet, genes encoding for essential proteins might be inactivated in specific conditions or their inactivation can be compensated by secondary mutations [13]. This is the case for c-di-AMP synthesis in *Listeria monocytogenes*, in which spontaneous mutations in genes involved in central metabolism and in adaptation to starvation allow growth without c-di-AMP [14]. Accordingly, c-di-AMP synthesis was shown to be dispensable for growth on minimal media by limiting the downstream effect of the (p)ppGpp alarmone on the global regulator CodY [14]. Additionally, spontaneous mutations in pyruvate carboxylase (PycA), an enzyme of the tricarboxylic acid (TCA) cycle, also lead to a toxic accumulation of metabolites in the absence of c-di-AMP in several lactic acid bacteria [15–17]. However, the compensatory mechanism appears distinct in other bacteria. In *Bacillus subtilis*, c-di-AMP synthesis is essential in rich media [18, 19], but the absence of c-di-AMP synthesis can be compensated by spontaneous mutations leading to an increased activity of the NhaK cation/proton antiporter allowing to overcome potassium toxicity [20]. In *Staphylococcus aureus*, c-di-AMP synthesis becomes dispensable when accumulating mutations in amino acid and osmolyte transporters, as well as through mutations in genes encoding for proteins required for respiration, linking c-di-AMP essentiality with osmoregulation and metabolism [21].

Furthermore, it has been shown that c-di-AMP binds to and regulates protein activities or riboswitches [12]. Notably, several RCK_C domain (regulator of conductance of K^+^)-containing proteins bind c-di-AMP [22]. RCK_C domains are present mainly in Ktr/Trk potassium transporter family proteins and c-di-AMP negatively regulates their transporter activities in different species [22–25]. C-di-AMP also often binds to and regulates the activity of CBS (cystathionine-ß-synthase) domains, a widespread nucleotide binding domain [26] present in osmoprotectant transporters, such as in OpuCA homologues [27, 28], and in proteins of unknown function [16]. Osmoprotectants, such as glycine betaine or carnitine, are compatible solutes, which are necessary together with potassium, to tolerate hyperosmotic shock [29, 30]. The KdpDE two-component system of *S*. *aureus* [31] and the *ydaO* riboswitch in *B*. *subtilis* [20, 32] bind c-di-AMP to control the expression of potassium transporters [20, 32]. Direct regulation of the pyruvate carboxylase activity by c-di-AMP in *L*. *monocytogenes* might also be related to intracellular potassium homeostasis through TCA-dependent accumulation of glutamate acting as a counterion of potassium [15, 16, 33].

In this study, we have characterized the ‘essential’ c-di-AMP function in *Streptococcus agalactiae* (the Group B *Streptococcus*, GBS), the main etiological agent of bacterial invasive infection in neonates [34]. GBS synthesizes and releases c-di-AMP in infected macrophages, but limits its detection by the host immune system by degrading extracellular c-di-AMP with a cell wall-anchored ectonucleotidase [10]. By analysing c-di-AMP synthesis in GBS, we report here that osmotic homeostasis is the critical cellular function regulated by c-di-AMP. The main mechanism involves binding of c-di-AMP to the transcription factor BusR which negatively regulates the expression of the *busAB* operon encoding for the glycine betaine BusAB transporter. Overall, c-di-AMP-dependent regulation of potassium and compatible solute transporters is conserved, but specific mechanisms of osmoregulation are present in each species and c-di-AMP also regulates these species-specific mechanisms to remain a central osmoregulator.

## Results

### c-di-AMP synthesis is essential under standard growth conditions

**Fig 1:**
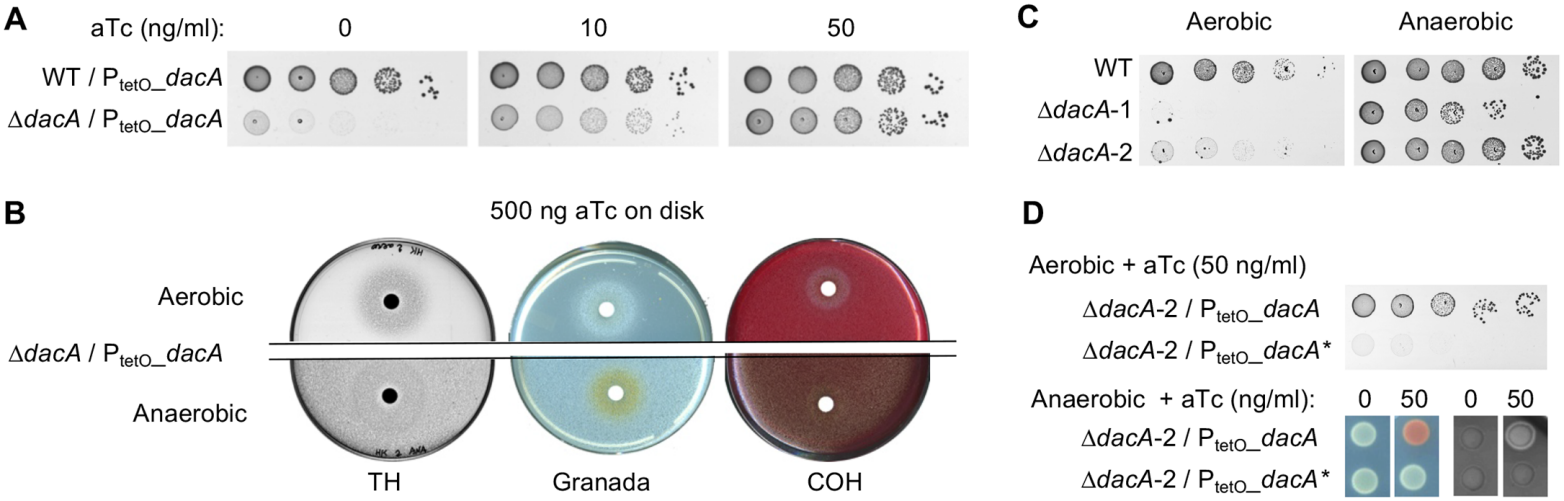
c-di-AMP synthesis is conditionally essential in GBS. (A) Serial dilutions of the WT and Δ*dacA* mutant containing the inducible P_tetO__*dacA* vector were spotted on TH agar supplemented or not with anydrotetracycline (aTc), and incubated 24 hours in standard growth condition (*i*.*e*. aerobic condition). (B) Growth of the Δ*dacA* / P_tetO__*dacA* mutant on TH, Granada, and Columbia + 5% horse blood (COH) incubated in aerobiosis or anaerobiosis for 24 hours. About 10^4^ bacteria were spread on each media and a disk containing 500 ng aTc was added on each plate before incubation. (C) Growth of the Δ*dacA*-1 and Δ*dacA*-2 mutants on TH incubated in aerobiosis or anaerobiosis. (D) Growth of the Δ*dacA*-2 mutant complemented with the WT *dacA* or the inactivated dacA* alleles under the control of the inducible promoter P_tetO_ on TH incubated in aerobiosis with aTc, and on Granada and COH in anaerobiosis with or without aTc.

In the GBS genome, a single gene, thereafter named *dacA*, encodes a protein containing a DisA_N domain (PF02457 Pfam domain), the only known domain with c-di-AMP synthesis activity [11]. The *dacA* gene is localized in a highly conserved three-gene operon encoding DacA, a putative DacA activity regulator (Gbs0903) and the essential GlmM enzyme (Gbs0904*)* involved in synthesis of cell-wall metabolite precursors [9, 11, 18, 19]. All attempts to inactivate *dacA* using standard protocols were unsuccessful, suggesting that *dacA* is an essential gene. Therefore, a conditional Δ*dacA* mutant was constructed in a strain bearing an ectopic copy of *dacA* cloned on a replicative vector and transcribed from the anhydrotetracycline (aTc)-inducible promoter P_tetO_ (S1 Fig). The growth of the Δ*dacA* / P_tetO__*dacA* mutant is aTc dose-dependent on TH medium incubated in aerobic growth conditions (Fig 1A). The mutant does not grow in the absence of aTc, while its growth was similar to that of the WT strain in presence of 50 ng/ml aTc.

By testing Granada medium, a GBS-specific medium developed to detect the orange-red polyenic pigment granadaene under anaerobic conditions [35], we unexpectedly observed growth of the Δ*dacA* / P_tetO__*dacA* mutant in the absence of aTc (Fig 1B). This anaerobic growth is independent from the medium components since it was also observed when grown in TH or Columbia Horse Blood (COH) agar. In contrast, the growth is aTc-dependent in aerobic condition whatever medium used (Fig 1B). In addition, Δ*dacA* / P_tetO__*dacA* colonies are not pigmented and not hemolytic in anaerobic conditions, unless the aTc-dependent ectopic *dacA* copy was expressed (Fig 1B). This indicates that c-di-AMP synthesis is necessary for granadene production, the GBS pigment that is also a ß-hemolysin/cytolysin [36].

The anaerobic growth of the Δ*dacA* / P_tetO__*dacA* strain was exploited to construct Δ*dacA* mutants without an ectopic *dacA* allele. The first Δ*dacA*-1 mutant was selected after anaerobic growth following the loss of the vector containing the additional *dacA* copy (S1 Fig). The second Δ*dacA*-2 mutant was constructed from the parental Δ*dacA*::*dacA* integrant by selecting the deletion mutant directly under anaerobic conditions (S1 Fig). Both Δ*dacA* mutants grow in anaerobiosis, although the Δ*dacA*-1 mutant growth is slightly altered on TH compared to the WT strain, and they do not grow in aerobiosis (Fig 1C). Re-introduction of the P_tetO__*dacA* vector in the two Δ*dacA* mutants restored growth, pigmentation, and hemolysis in the presence of aTc (Fig 1D). In contrast, expression of an inactivated DacA*, bearing a R_213_K substitution in the RHR conserved di-adenylate cyclase motif [37, 38], does not complement the Δ*dacA* phenotypes (Fig 1D). As expected, the purified recombinant DacA protein produces c-di-AMP from two molecules of ATP while the recombinant DacA* is devoid of di-adenylate cyclase activity (S2 Fig). Thus, c-di-AMP synthesis appears essential for growth in aerobiosis and necessary for optimal growth in anaerobiosis.

### Mutation of the BusAB transporter is necessary in the absence of c-di-AMP

The genomes of the parental WT NEM316 strain, of the two Δ*dacA*::*dacA* integrants, and of the two corresponding Δ*dacA* mutants were sequenced (S1 Table). Compared to the published reference sequence [39] (RefSeq NCBI NC_004361), fifteen SNPs or INDELs are present in our WT strain and in all of its progeny (S2 Table). The genome sequence of the first Δ*dacA*::*dacA* integrant is identical to the parental WT strain, while the second integrant displays a SNP located in the *cylD* gene of the *cyl* operon encoding the ß-hemolysin/cytolysin [36, 40] (S3 Table).

**Fig 2:**
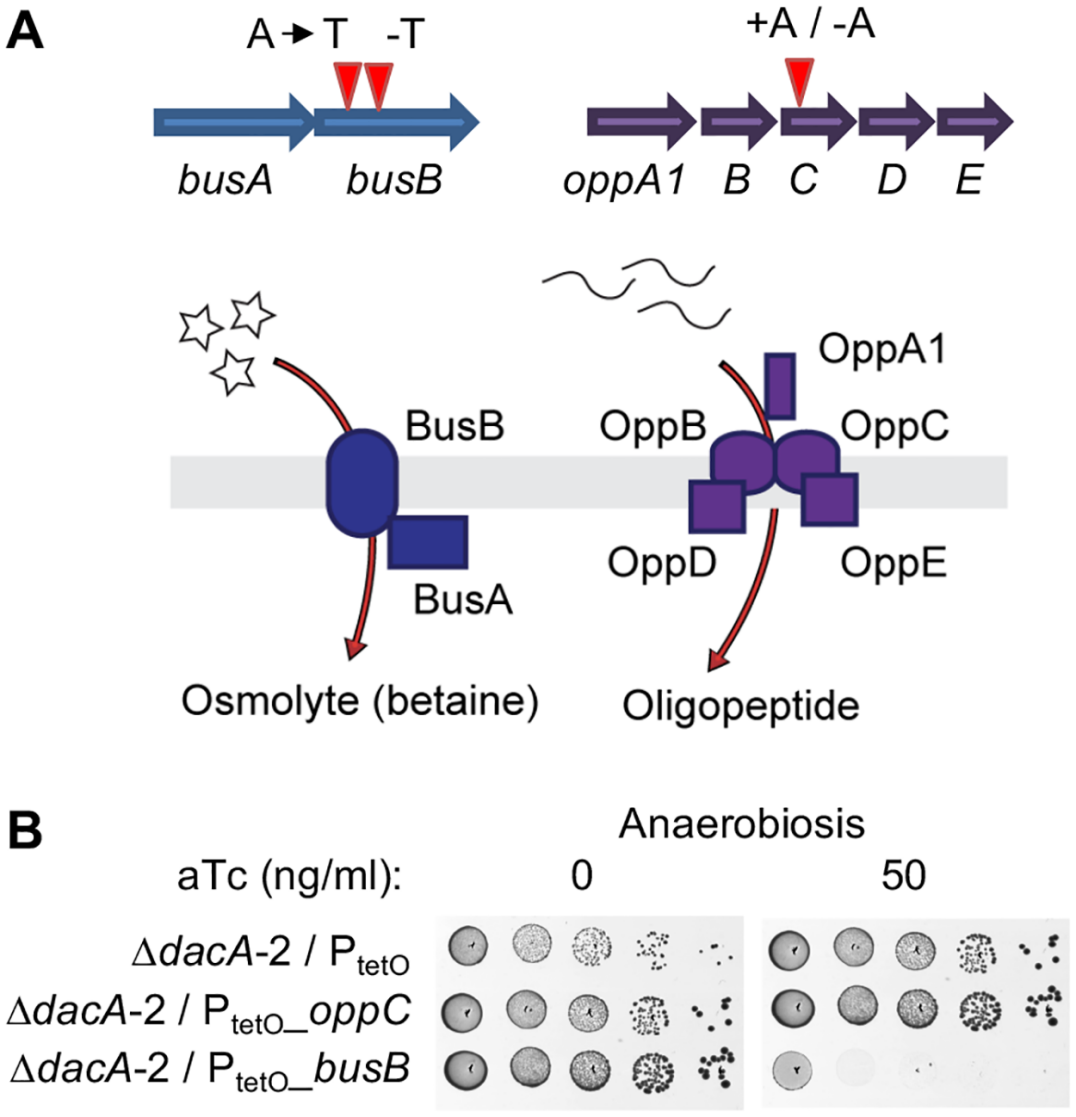
Mutation of the BusAB transporter is necessary in the absence of c-di-AMP. (A) Schematic representations of the independent *busB* and *oppC* mutations in Δ*dacA* mutants and of the BusAB and Opp transporters. (B) Conditional expression of a WT copy of *oppC* and *busB* in the Δ*dacA*-2 mutant in anaerobiosis on TH with and without aTc. The Δ*dacA*-2 mutant with the empty vector P_tetO_ is used as a control.

Compared to their parental integrants, the two Δ*dacA* mutants have two additional mutations in the same genes: *oppC* (the gbs0146 locus) and *busB* (the gbs1838 locus) (Fig 2A and S3 Table). The first gene encodes the OppC oligopeptide transporter subunit [41] and the two mutants have independent frameshift mutations (+A in Δ*dacA*-1 and—A in Δ*dacA*-2) located at the beginning of the gene (Fig 2A). The second gene encodes a transmembrane protein homologous to the *Lactococcus lactis* BusB subunit [42]. In this species, BusB and its cytoplasmic partner BusA form an ABC transporter involved in osmolyte import (Fig 2A). In the Δ*dacA* mutants, *busB* has either a SNP resulting in a V_62_D substitution localized in the first transmembrane domain of BusB (Δ*dacA*-1) or a single nucleotide deletion at position 120 (Δ*dacA*-2) (S3 Table). In addition to the *busB* and *oppC* mutations, the Δ*dacA*-1 mutant has an additional copy of TnGBS, a 47kb integrative and conjugative element already present three times in the parental strain [39, 43], integrated in an intergenic region (S3 Table).

To assess the functional significance of the two shared mutated genes, we introduced a replicative vector containing a wild-type copy of *oppC* or *busB* under the control of the aTc inducible P_tetO_ promoter in the Δ*dacA* mutants (Fig 2B). Expression of a WT copy of *busB*, but not of *oppC*, inhibited the anaerobic growth of the Δ*dacA* mutants (Fig 2B). Therefore, a mutation in the osmolyte transporter BusB appears necessary to counteract the effect of *dacA* inactivation under anaerobic conditions. The independent occurrence of a loss of function mutation in *oppC* in the two mutants also suggested that this mutation was necessary but not sufficient.

### Adaptation to the absence of c-di-AMP synthesis involves intertwined mutations

**Fig 3:**
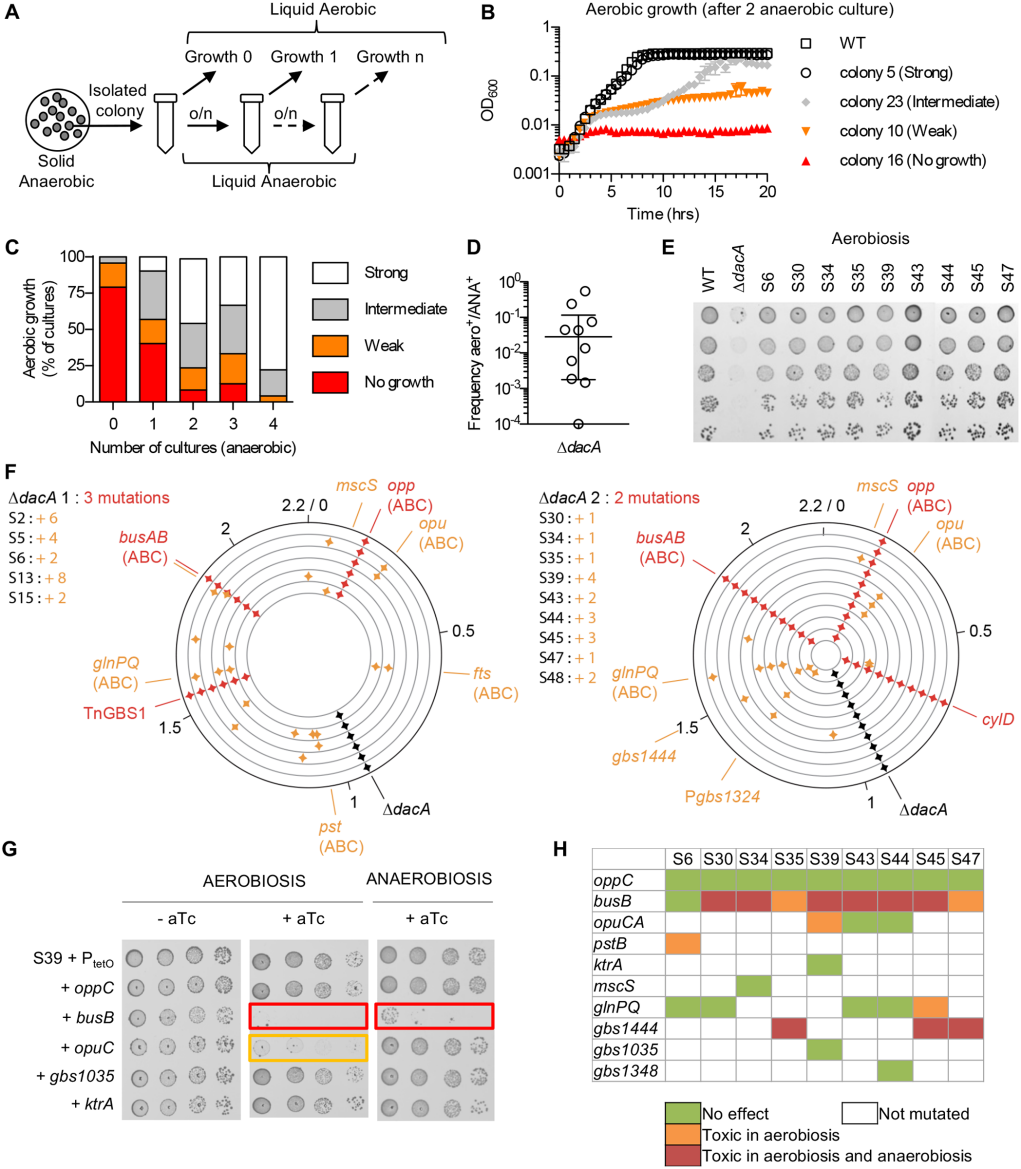
Adaptation to the absence of c-di-AMP synthesis involves intertwined mutations. (A) Schematic representation of the experiment. Strains were propagated on solid media in anaerobiosis. Isolated colonies were picked in TH and incubated overnight (o/n) at 37°C in anaerobiosis. At each serial dilution step (0, 1, … n), aliquots were taken and the growth in aerobiosis was monitored in triplicate. (B) Representative growth curves of the Δ*dacA*-2 mutant in aerobiosis after two serial culture in anaerobiosis. The growth curves obtained from 4 independent Δ*dacA* colonies (number 5, 8, 9 and 16) illustrate the variability. Growth curves were classified as corresponding to no growth (OD < 0.01), weak (OD < 0.1), intermediate (OD > 0.1 at 20 hrs), or strong adaptation (OD > 0.1 at 10 hrs). Growth curves are the mean of triplicate +/- SEM for each independent colony. (C) Percentage of Δ*dacA*-2 cultures (n = 24) showing aerobic adaptation following serial cultures in anaerobic condition. Similar results were obtained with 24 cultures done with the Δ*dacA*-1 mutant. (D) Frequency of Δ*dacA* mutant able to grow in aerobiosis on TH agar plates after dilution of one overnight anaerobic cultures. Median with interquartile range were calculated from ten independent cultures. (E) Suppressors corresponding to Δ*dacA* mutant able to grow aerobically were isolated and their phenotype confirmed by spotting on TH agar. (F) Distribution of the mutation on the 2.2 Mb genome of Δ*dacA*-1 (left) and Δ*dacA*-2 (right) mutants (outer ring) and of Δ*dacA* suppressors (inner rings). The *dacA* deletion is highlighted in black. Mutations in the Δ*dacA*-1 and Δ*dacA*-2 mutants absent in the parental WT strain are color-coded in red, and mutations specific of suppressors are in orange. Identity of gene or operon independently mutated in more than two strains are shown. ABC transporters are indicated in bracket. (G) Conditional expression of a WT copy of mutated genes in the Δ*dacA* suppressors S39. Phenotypic effect of the expression of each gene was tested by adding aTc (50 ng/ml) in TH. Coloured boxes highlight growth inhibition upon expression of a WT allele in aerobiosis and anaerobiosis (red boxes), or aerobiosis only (orange). (H) Same experiment as in (G) in 9 Δ*dacA* suppressors. See S3 Fig for the corresponding images.

Attempts to delete *dacA* in Δ*busB*, Δ*oppC*, and Δ*busB* Δ*oppC* backgrounds were unsuccessful, suggesting the necessity of additional compensatory mutations. To identify these additional pathways, we selected Δ*dacA* clones able to grow in aerobiosis. In liquid cultures, the Δ*dacA* mutants display high growth variability that was recorded by following their aerobic growth in liquid medium (Fig 3A). When isolated colonies (n = 48) grown anaerobically were directly inoculated in liquid media, around 75% were unable to grow under aerobic conditions, the remaining cultures showing weak or intermediate growth defects (Fig 3B and 3C). However, after 4 serial cultures under anaerobic conditions, almost three quarters of these cultures were able to grow as the WT strain under aerobic conditions (Fig 3C). The growth of each culture remains highly variable, suggesting that different populations arose and co-exist during serial cultures. However, this is not due to a higher mutation rate of the Δ*dacA* mutants since rifampicin resistant colonies were obtained at a similar frequency with WT and Δ*dacA* mutant strains (S2 Fig).

Fourteen independent Δ*dacA* suppressors (5 from Δ*dacA*-1 and 9 from Δ*dac*-2) were isolated on TH in aerobiosis after a single overnight incubation in liquid medium in anaerobiosis. In this condition, the proportion of colonies growing on TH in aerobiosis is highly variable, usually between 0.5 and 10^−3^ (Fig 3D). Each isolated suppressor grew on TH plates as the WT (Fig 3E), and the absence of c-di-AMP in whole bacterial extracts of Δ*dacA* mutants and of several suppressors was confirmed (S2 Fig), excluding that a cryptic di-adenylate cyclase was activated to compensate for the absence of *dacA*.

The genomes of the 14 Δ*dacA* suppressor strains were sequenced to identify the compensatory mechanisms, but the number of mutations were variable, with no single mutated gene common to all suppressors (S3 Table). All suppressors carry the two *oppC* and *busB* mutations present in the parental Δ*dacA* mutant and between 1 to 7 additional mutations (Fig 3F). The mutations are mostly SNPs (n = 24, including 19 leading to amino-acid substitution), followed by small indels (n = 12, including 8 in coding sequence), three deletions of 36–47 bp, and one 90-bp duplication (S3 Table). Independent mutations in the same gene or functional complex were identified in different suppressors (Fig 3F), including mutations in an operon encoding a second osmolyte ABC transporter homologous to the *L*. *lactis* OpuABC glycine betaine transporter [44], in the glutamine ABC transporter GlnPQ [45, 46], and in a putative secreted protein (Gbs1444) of unknown function (S4 Table). Also interesting is the presence of additional loss of function mutations in BusB in suppressor S5 originating from the Δ*dacA*-1 mutant with the BusB V_62_D substitution (S3 Table).

To identify causative mutations restoring growth in the absence of c-di-AMP, we focused our analysis on nine different suppressor mutants. In each of these suppressors, a WT copy of the mutated genes expressed from the aTc-inducible promoter was introduced. As expected, induction of the WT copy of *busB* inhibited the growth of six of the nine suppressors in aerobiosis and anaerobiosis (Fig 3G and 3H, and S3A Fig). The expression of a WT *busB* allele was toxic only under aerobic growth in two suppressor mutants, and had no effect on one suppressor (Fig 3H). These results confirm that *busB* inactivation is necessary for bacterial growth in the absence of *dacA*, but reveal that additional mutations can alleviate *busB* toxicity in the absence of c-di-AMP.

Among the eight genes mutated at least once in the nine suppressors, four are toxic upon re-expression of their WT copy in five of the suppressors (Fig 3H and S3A Fig). The expression of Gbs1444 encoding a putative secreted protein is toxic in the three suppressors containing a mutation in this gene. The remaining three genes inhibiting growth upon their re-expression encode for ABC transporters: OpuCA, GlnPQ, and PstB (a phosphate ABC transporter homolog). We excluded a non-specific toxic effect of the tested genes by expressing them in the same condition in a WT background (S3B Fig).

Overall, at least one mutated gene in each of the nine suppressors studied was toxic upon conditional expression of a WT copy, suggesting that the corresponding mutation compensates the absence of c-di-AMP. Nevertheless, the pattern of mutations suggests strong epistasis, *i*.*e*. the effect of the toxic gene is dependent of the other mutations present in a given suppressor. For instance, in suppressors S30 and S34, mutation of *busB* is not sufficient for aerobic growth of the parental Δ*dacA* mutants, but should be combined with *glnPQ* or *mscS* (Fig 3H). In contrast, in suppressors S6, S35, and S47, the toxic effect of a functional *busB* allele in a Δ*dacA* background can be attenuated by mutations in *gbs1444* or *pstB* (Fig 3H). The pattern of compensatory mutations and epistatic interactions suggest that c-di-AMP controls a highly regulated and interconnected essential pathway.

### c-di-AMP binds conserved osmolyte and potassium transporters

Compensatory mutations might encode for proteins directly regulated by c-di-AMP. To identify these direct c-di-AMP regulated processes, interaction between c-di-AMP and candidate proteins were assayed by DRaCALA [47]. Fourteen proteins were selected as candidates, including the BusA, OppD, and OppE cytoplasmic ATPases subunits of osmoprotectant and oligopeptide transporters (Fig 2), the mutated proteins tested for their phenotypes upon re-expression in Δ*dacA* suppressors (Fig 3H), and three additional proteins containing a RCK_C/TrkA_C domain with a putative c-di-AMP binding motif [22–24]. The corresponding genes were cloned and expressed as a fusion protein in *E*. *coli* (S4 Fig), and whole-cell extracts were incubated with radiolabelled c-di-AMP.

**Fig 4:**
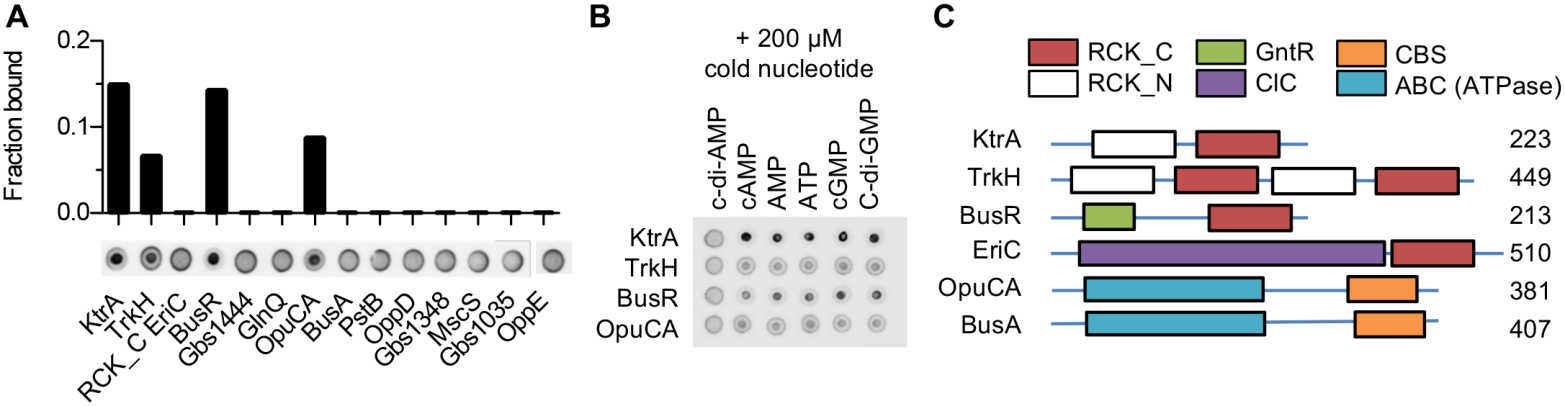
c-di-AMP binds three transporters subunits and a transcriptional factor. (A) Interaction of radiolabelled c-di-AMP with targeted protein by DRaCALA. Full-length proteins were expressed in *E*. *coli*, except for EriC where only the RCK_C can be expressed. Whole *E*. *coli* extracts were mixed with radiolabelled c-di-AMP and spotted on a nitrocellulose membrane. C-di-AMP bounds to protein does not diffuse as far as free c-di-AMP. Quantification of the inner and outer circles intensities allows to calculate the fraction of bound c-di-AMP. (B) Specificity of the c-di-AMP interaction. Same as (A) with the addition of cold competitor to the reaction before spotting on membrane. (C) Color-coded representation of the domain organisation of selected proteins. Number of amino acids are indicated at the end of proteins. The RCK_C (red) and CBS (orange) domains are predicted c-di-AMP and nucleotides binding domains, respectively. The RCK_N domain (regulator of potassium conductance, white) is prevalent among potassium channels. The GntR domain (green) is a winged helix-turn-helix DNA binding domain. The ABC domain (blue) represent the ATPase domain of ABC transporter. The ClC domain (purple) is found in chloride ion channels, a family of voltage-dependent gating transporter with 11 transmembrane domains.

C-di-AMP binds to four proteins: KtrA (Gbs1678), TrkH (Gbs1639), OpuCA (Gbs0235) and Gbs1201, thereafter named BusR (Fig 4A). The binding of radiolabelled c-di-AMP is specific since it could be displaced by addition of cold c-di-AMP but not of c-di-GMP, cAMP, cGMP, AMP or ATP (Fig 4B). Three of the four c-di-AMP binding proteins are homologs of conserved potassium (KtrA and TrkH) and osmolyte (OpuCA) transporters. Two of them, KtrA and OpuCA, are mutated in one or three of the nine suppressors, respectively (S4 Table). These two proteins are conserved c-di-AMP binding proteins, where binding is dependent on their RCK_C/TrkA_C [22, 23] or CBS [27, 28] domains, respectively. Among the four GBS proteins containing RCK_C/TrkA_C domains tested (Fig 4C), only one, EriC (Gbs1174), a chloride channel homolog, did not give a positive signal with c-di-AMP in our DRaCALA screen (Fig 4A). However, only the RCK_C domain of EriC was used in this experiment (S4 Fig) as we failed to express in *E*. *coli* the full-length protein with its eleven transmembrane domains. Therefore, these results do not rule out the possibility that a full length EriC might bind c-di-AMP.

It is also interesting to note that BusA and OpuCA are two highly similar subunits of osmolyte transporters (55% similarities, e = 6 e-59). The two proteins contain a CBS domain (Fig 4C). However, BusA, the cytoplasmic subunit of the BusAB transporter which is mutated in Δ*dacA* mutants, does not bind c-di-AMP in contrast to OpuCA (Fig 4A). This confirms that CBS domains may have a similar topology but different physiological ligands [26]. This also implies different mechanisms of regulation for the BusAB and OpuC osmolyte transporters.

### BusR directly represses the *busAB* transporter necessary for c-di-AMP dependent osmotic regulation

The fourth c-di-AMP binding protein identified by DRaCALA is a putative transcriptional regulator of the GntR family containing a winged helix-turn-helix DNA binding domain (Fig 4C). BusR is highly similar to the annotated MngR trehalose transcriptional repressor in *Chlamydia trachomatis* (e value = 2 e-107) and to the *L*. *lactis* BusR transcriptional repressor (5 e-61). In *L*. *lactis*, the *busR* gene is localized immediately upstream of the *busAB* operon [48], whereas in GBS *busR* and *busAB* are separated by 655 kb and no transcriptional regulator is located in the vicinity of the *busAB* operon.

**Fig 5:**
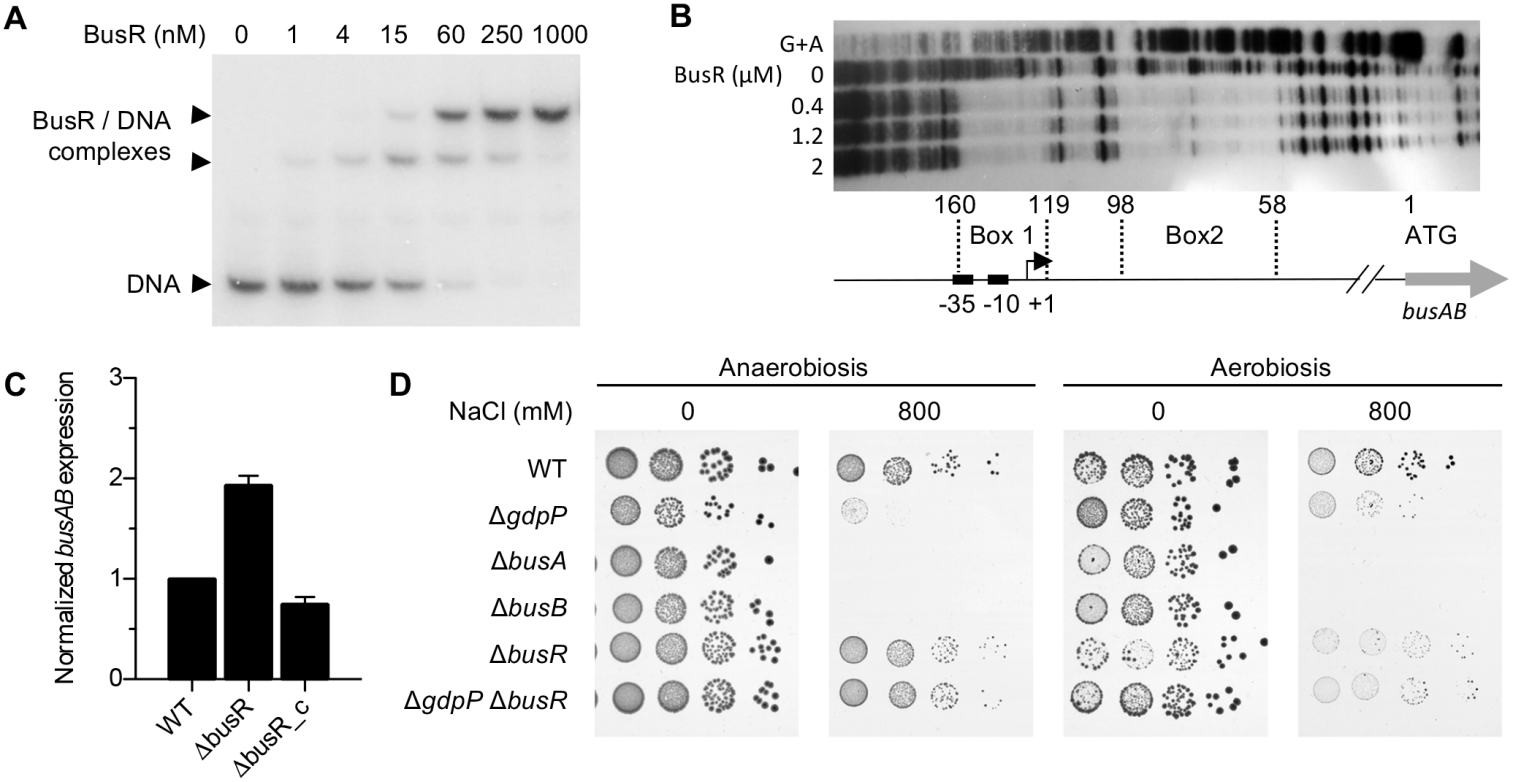
BusR is a transcriptional repressor of the osmolyte transporter BusAB. (A) Gel shift assay with increasing concentration of recombinant BusR and the radiolabelled P_*busAB*_ promoter. (B) Footprint experiment on the P_*busAB*_ promoter with BusR. The two DNase I protected boxes are numbered from the start codon of the *busAB* operon. The position of the transcriptional start site and of the -10 and -35 elements are highlighted. (C) Quantification of *busAB* transcript by RT-qPCR in the WT, the Δ*busR* mutant, and the Δ*busR*_c complemented strain. Means and SD are calculated from three independent RNA purification done from exponentially growing cultures in TH. (D) Spotting dilutions of WT, Δ*gdpP*, Δ*busA*, Δ*busB*, Δ*busR*, and Δ*gdpP* Δ*busR* cultures on TH with and without 800 mM NaCl incubated in aerobiosis and anaerobiosis.

The homology with *L*. *lactis* suggests a putative conserved function of BusR on *busAB* transcription in GBS. Therefore, we purified recombinant GBS BusR and tested its binding on the P_*busAB*_ promoter of the *busAB* operon. Gel shift assays show that P_*busAB*_ migrates more slowly in the presence of BusR (Fig 5A) and footprint experiments show two BusR-protected regions in the P_*busAB*_ promoter, one overlapping the -35 and -10 elements and the +1 transcription start site (Fig 5B). Deletion of *busR* increases expression of the *busAB* operon compared to the WT or the Δ*busR*_c complemented strain (Fig 5C). These results demonstrated that the c-di-AMP binding protein BusR is a transcriptional regulator directly repressing the *busAB* operon.

To test the functional link between c-di-AMP and the BusR-BusAB osmolyte import system, we analysed the phenotypes of the deletion mutants (Δ*busA*, Δ*busB*, Δ*busR*, Δ*gdpP*, and Δ*busR* Δ*gdpP*) in response to osmotic stresses. As observed in several bacteria, deletion of the c-di-AMP phosphodiesterase GdpP increases the intracellular c-di-AMP concentration in GBS (20- to 38-fold, S2C Fig). Furthermore, the Δ*gdpP* mutant is more susceptible to hyperosmotic stress compared to the WT strain (Fig 5D). Strikingly, Δ*gdpP* osmo-susceptibility is dependent on a functional BusR transcriptional regulator. Deletion of BusR has no or a weak effect on bacterial growth upon hyperosmotic challenge, while the two subunits of the BusAB transporter are as important as GdpP to resist the hyperosmotic stress (Fig 5D). The double Δ*busR* Δ*gdpP* deletion abolishes the susceptibility of the Δ*gdpP* mutant (Fig 5D), showing that elevated c-di-AMP leads to hyperosmotic susceptibility by acting through the transcriptional repressor BusR.

### c-di-AMP is dispensable for growth in osmolyte-depleted media

**Fig 6:**
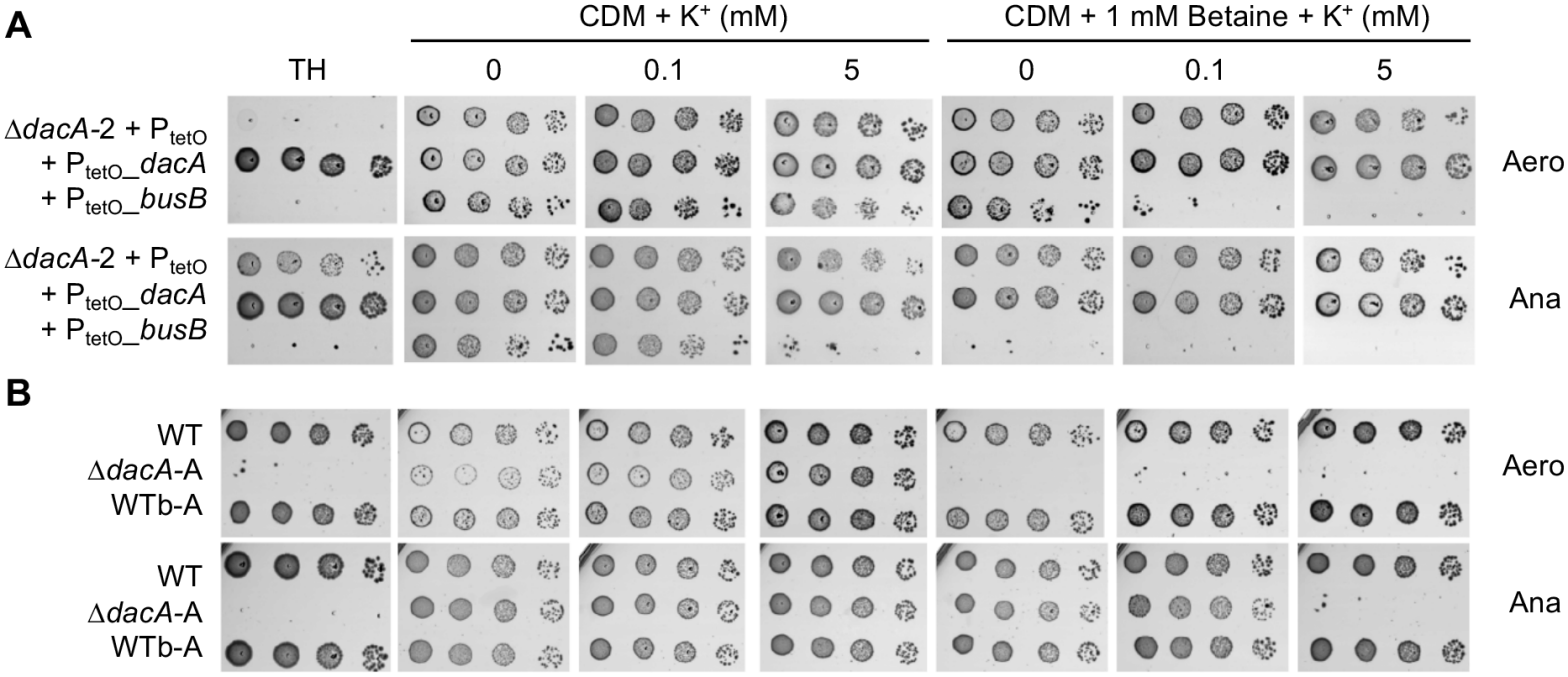
c-di-AMP is dispensable in osmolyte depleted medium. (A) Growth of the Δ*dacA*-2 mutant with the empty vector control (P_tetO_), and the *dacA* or *busB* conditional expression vectors (P_tetO__*dacA* or P_tetO__*busB*) on media with 50 ng/ml aTc. The rich TH medium was used as control and potassium and glycine betaine was added to synthetic medium (CDM) incubated in aerobiosis and anaerobiosis. (B) Same experiment as in (A) with the Δ*dacA*-A mutant and its isogenic WTb-A control.

The pattern of compensatory mutations and the identification of c-di-AMP binding proteins point towards a coordinated regulation of potassium and osmolyte uptake as the essential function of c-di-AMP in GBS. We therefore tested the growth of the Δ*dacA* mutant in a chemically defined medium (CDM) with variable potassium and osmolyte concentrations (S5 Table). To this end, we used the Δ*dacA-2* mutant with an empty vector, a *dacA* complementing vector, or a *busB* expressing vector to complement the *busB* loss-of-function mutation in this mutant. In this CDM, c-di-AMP synthesis is dispensable for bacterial growth regardless of the potassium concentration and incubation condition, except for anaerobic growth of the mutant expressing a WT copy of *busB* at high potassium concentrations (5 mM) (Fig 6A). Strikingly, addition of glycine betaine to CDM inhibits the growth of the Δ*dacA-2* mutant expressing *busB* regardless the potassium concentration, except in aerobiosis at extremely low concentrations of potassium (Fig 6A).

The inhibitory effect of glycine betaine is dependent on *busB* expression since glycine betaine does not inhibit the growth of the Δ*dacA-2* mutant with the empty vector (Fig 6A) and has no effect on the Δ*dacA-2* / P_tetO__*busB* mutant in the absence of aTc. Similarly, the inhibitory effect of glycine betaine is observed with carnitine, a related osmolyte [49], while choline, a common precursor of glycine betaine, has no effect (S5 Fig). In the same conditions, *busB* expression in a WT strain has no effect on growth (S5 Fig), showing that osmolytes such as glycine betaine or carnitine need the expression of *busAB* and the absence of c-di-AMP to be toxic. Overall, the presence of an osmolyte in the culture medium appears to be the main cause of growth inhibition in the absence of c-di-AMP synthesis. The concentration of potassium is also important under specific conditions ([K^+^]_high_ in anaerobiosis and [K^+^]_low_ in aerobiosis in presence of osmolyte), suggesting that growth inhibition results from a combination of dysregulated potassium and osmolyte uptake.

### c-di-AMP is essential to avoid the inhibitory effect of osmolytes

To test if the growth condition is sufficient to alleviate the essential function of *dacA*, we repeated the construction of a Δ*dacA* mutant except that all steps were performed in CDM without osmolyte and with 0.5 mM potassium. In this condition, we readily obtained Δ*dacA* mutants and their respective WTb controls at high frequency (S1 Fig). On CDM, the growth of the new Δ*dacA*-A mutant was similar to the WT and WTb controls regardless the potassium concentration and incubation conditions (Fig 6B). Addition of glycine betaine inhibits Δ*dacA*-A at all tested potassium concentrations in aerobic condition and only at high potassium concentration in anaerobic condition (Fig 6B). Finally, the Δ*dacA*-A mutant was unable to grow on TH (Fig 6B). Two additional Δ*dacA* mutants (-B and–C), obtained from independent parental Δ*dacA*::*dacA* integrants, displayed the same phenotypes as the Δ*dacA*-A mutant. These results confirmed that c-di-AMP synthesis is essential in rich medium and dispensable in minimal medium, unless osmolytes are present. The inhibiting effect of osmolytes is dependent on aerobiosis and anaerobiosis and, to a lesser extent, on potassium concentrations, suggesting a link between osmotic regulation and metabolism.

The genome of the three new, independent pairs of Δ*dacA* and WTb strains were sequenced (S1 Table). None of the Δ*dacA*-A to -C mutants share a mutation with the previously sequenced Δ*dacA-*1, Δ*dacA*-2, and Δ*dacA* suppressors (S3 Table). The only exception is the *cylD* SNP in the Δ*dacA*-B that is also present in the Δ*dacA*-2 mutant and their common parental Δ*dacA*::*dacA* integrant (S3 Table). Still, the three Δ*dacA*-A to–C mutants each have one mutation compared to the WT strain. These mutations are localized in *gbs0330*, encoding the transcriptional repressor FabT (S3 Table), embedded in the *fab* operon encoding enzymes of the essential type II fatty acid synthesis pathway [50]. Unexpectedly, the WTb controls and two of the three parental Δ*dacA*::*dacA* integrants show the same *fabT* mutations (S3 Table). The independent *fabT* mutations imply a strong selective pressure most probably due to the nutritional supply in the medium and not to c-di-AMP depletion. Targeted sequencing of the *fabT* locus of the WT and Δ*dacA*::*dacA* integrants after growth in overnight cultures in TH and CDM 0.5 mM K^+^ confirmed that *fabT* mutations are selected at a high frequency only on CDM medium independently of c-di-AMP (S6 Fig).

## Discussion

**Fig 7:**
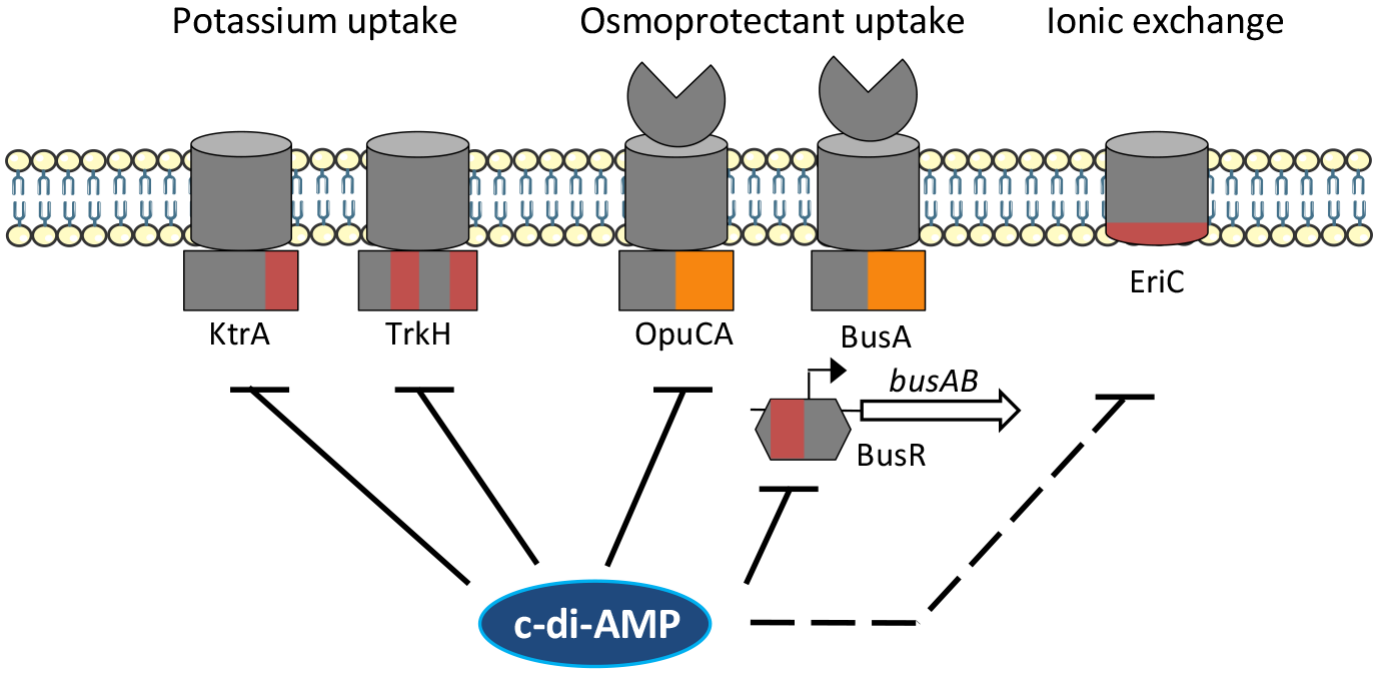
c-di-AMP is a central regulator of osmotic homeostasis in GBS. Coordination of osmotic transporters by c-di-AMP occurs at the post-translational and transcriptional levels. The KtrA and TrkH potassium transporter subunits and the OpuCA osmolyte transporter subunit are conserved c-di-AMP binding proteins. The c-di-AMP binding BusR transcriptional factor is a repressor of the second osmolyte transporter BusAB. Inactivation of BusR leads to *busAB* expression, a main cause of growth inhibition in the absence of c-di-AMP in rich media or in presence of osmolytes. C-di-AMP might also regulate EriC, a RCK_C domain containing chloride channel protein with 11 transmembrane domains. The RCK_C and CBS domains are color-coded red and orange, respectively.

Here we demonstrate that the essential function of c-di-AMP in *S*. *agalactiae* is to regulate osmotic homeostasis. The mechanism involves the conserved binding of c-di-AMP to potassium and osmoprotectant transporters (Ktr, Trk, OpuC) and the BusR c-di-AMP binding transcriptional regulator controlling the transcription of the *busAB* operon encoding the BusAB osmoprotectant transporter (Fig 7). Our study strengthens the recent proposal that c-di-AMP has a conserved and essential role in maintaining osmotic homeostasis in Gram-positive bacteria [51]. Typically, osmoregulation is achieved through three conserved processes: a rapid potassium uptake, the synthesis or import of compatible solutes, and a final ionic exchange to restore the membrane potential [29, 30]. However, each bacterial species encodes a different set of functionally related transporters and has evolved specific regulatory mechanisms, probably a consequence of the long-term adaptation of the bacteria to their environments [52–54]. Notwithstanding this evolution, c-di-AMP preserves its role in regulating core components of the osmotic response while adapting to control the species-specific transporters and regulators.

Direct inhibition of potassium transporters containing a RCK_C domain is a conserved mechanism of regulation exerted by c-di-AMP that is present in many bacteria [20, 22–24]. For example, such a coordinated regulation of potassium transporters, together with the regulation of the *ydaO* c-di-AMP riboswitch controlling the *kimA* gene encoding an additional high affinity potassium transporter, is essential in *B*. *subtilis* [20, 32]. Indeed, in the absence of c-di-AMP, the loss of transporters inhibition leads to a toxic accumulation of potassium, which can be bypassed by depleting potassium in the growth medium or by compensatory mutations increasing potassium efflux [20]. Differently to *B*. *subtilis*, we did not observe a strong effect of external potassium concentrations on the growth of *S*. *agalactiae* mutants unable to synthesize c-di-AMP, and we did not obtain compensatory mutations increasing potassium efflux, suggesting a different mechanism of regulation.

Indeed, we show here that the second step of the osmotic response, the uptake of compatible solutes, is the critical function regulated by c-di-AMP in *S*. *agalactiae*. These compatible solutes are necessary to equilibrate the osmotic pressure and to avoid the deleterious consequences of potassium uptake on metabolism. This regulation involves c-di-AMP binding to the OpuC glycine betaine transporter, which is conserved in several species, including *S*. *aureus* [27] and *L*. *monocytogenes* [28]. As we observed in *S*. *agalactiae*, compensatory mutations have been obtained in osmoprotectant transporter encoding genes in *S*. *aureus* and *L*. *monocytogenes* [15, 21]. However, these mutations are not localized in the c-di-AMP binding protein OpuC homologues, but inactivated the highly similar *S*. *agalactiae* BusAB and *L*. *monocytogenes* Gbu [15] ABC transporters, or the *S*. *aureus* OpuD transporter belonging to the BCCT family [21]. In *S*. *agalactiae* and *S*. *aureus*, glycine betaine and related osmoprotectants inhibit the growth of diadenylate cyclase mutants, through the activity of the unrelated BusAB and OpuD transporters, respectively [21]. Therefore, the two species have evolved independent mechanisms allowing the essential regulation of compatible solute uptake by c-di-AMP.

In *S*. *agalactiae*, the transcriptional repressor BusR represents the link between c-di-AMP and BusAB as it controls the expression of the *busAB* operon. The BusR regulator belongs to the GntR family of proteins. It is not related to the only c-di-AMP binding transcriptional regulator characterized to date, the TetR-like DarR of *Mycobacterium smegmatis* [55]. Binding of c-di-AMP on BusR most probably involved its RCK_C regulatory domain which is present in a subset of GntR transcriptional regulators present mainly in streptococci, lactococci, and clostridi [56]. C-di-AMP regulation of transcription factors probably occurs in all these species, including the previously characterized *L*. *lactis* BusR whose binding on the promoter of *busAB* was demonstrated to be dependent on ionic strength [48, 57]. It is therefore likely that BusR homologues integrate c-di-AMP and intracellular potassium concentration to control gene transcription, but it remains to be determined whether these regulators control only genes involved in osmoregulation.

The unregulated import of osmolytes in the absence of c-di-AMP might inhibit growth as a consequence of cell poisoning, loss of membrane potential, or impaired cell division due to an incompatible internal osmotic pressure [52, 58]. The loss of osmotic homeostasis might be even exacerbated by a c-di-AMP regulation of ionic transporters such as the c-di-AMP binding cation/proton antiporter CpaA of *S*. *aureus* [22, 59] or the RCK_C domain containing chloride channel EriC of *S*. *agalactiae*. Indeed, to compensate the global dysregulation of osmotic systems, we observed several compensatory mutations in the *S*. *agalactiae* Δ*dacA* mutants, including one in the mechanosensitive channel protein MscS, a ion channel responding to membrane stress [60], and the GlnPQ amino acids [45, 46] and Opp oligopeptide [41, 61] ABC transporters. Notably, mutations in the oligopeptide transporter OppA-F and in the amino acid transporter AlsT are frequent in *L*. *monocytogenes* and *S*. *aureus* Δ*dacA* mutants [14, 15, 21]. In these two species, peptide and amino acid uptake is necessary to regulate their internal osmotic pressure, either directly or as precursors of osmoprotectants [15, 21]. The diversity of compensatory mutations in genes related to osmoregulation suggests that bacteria have different mechanism to restore an osmotic equilibrium to counterbalance potassium and osmoprotectant uptake in the absence of c-di-AMP.

It is noteworthy that in *S*. *agalactiae*, the growth of our initial Δ*dacA* mutants is oxygen-dependent. Interestingly, c-di-AMP synthesis is dispensable in *Streptococcus mutans* [62], which is routinely cultured under anaerobic conditions, and the link between oxygen and c-di-AMP synthesis was recently reported in *S*. *aureus* [21]. In this latter species, the growth inhibition of Δ*dacA* mutants in aerobiosis is not directly linked to respiration, but the respiratory chain must be inactivated to restore growth [21]. One hypothesis is that respiration is coupled to the TCA cycle, a central metabolic pathway in aerobiosis, which is critical for glutamate metabolism, and hence for osmoregulation [21]. Strikingly, pyruvate carboxylase, one of the key enzymes of the TCA cycle, is directly regulated by c-di-AMP in *L*. *monocytogenes* [15, 16]. In contrast, *S*. *agalactiae*, an aerotolerant anaerobe devoid of a functional TCA cycle [63, 64], is unable to respire unless an exogenous source of electron acceptors is provided. We observed that the difference between aerobic and anaerobic growth of the Δ*dacA* mutants in rich media is linked to the BusAB transporter, which suggests a differential regulation upon oxygen availability. Overall, bacteria might have adapted their mechanisms of osmoregulation to their metabolism and, probably, to their environment.

In conclusion, our study establishes c-di-AMP as an essential regulator of osmotic homeostasis in *S*. *agalactiae*. The main mechanism involves the c-di-AMP binding transcriptional regulator BusR that controls osmoprotectant uptake through the BusAB transporter. It is therefore likely that phylogenetically distant species have developed species-specific mechanisms to maintain their osmotic pressure while keeping c-di-AMP as the major coordinator of this essential cellular function. This functional conservation on a long evolutionary time-scale suggests that osmotic homeostasis is the main essential function regulated by c-di-AMP [33].

## Material and methods

### GBS strains and growth conditions

The WT GBS strain used in this study is NEM316, the originally sequenced (RefSeq NC_004368.1) serotype III reference isolate [39]. The usual Todd Hewitt (TH, Difco Laboratories), Columbia supplemented with 10% horse blood (BioMérieux), and Granada medium (BioMérieux) were used for propagation and phenotypic tests. A chemically defined medium (CDM) containing inorganic salts, vitamins, amino acids, nucleobases, pyruvate and glucose (S5 Table) was adapted from reference [65]. Glycine betaine, potassium chloride, and sodium chloride (Sigma-Aldrich) are added when stated. Buffering at pH 7.3 was done by adding Hepes (50 mM). Liquid GBS cultures are done in static condition incubated in aerobiosis or anaerobiosis. Anaerobiosis is obtained in hermetic jars with AnaeroGen gas packs (Oxoid, ThermoFischer). Growth curves in aerobiosis were done in 96 wells microplates (150 μl) at 37°C with constant shaking and automatic recording of OD_600_ every 20 minutes (BioTek Synergy). Erythromycin and kanamycin (Sigma-Aldrich) are used for plasmid selection at 10 and 500 μg/ml, respectively. Anhydrotetracycline (Sigma-Aldrich) is used for conditional expression from the P_tetO_ inducible promoter at 0–100 ng/ml [66]. Rifampicin (50 μg/ml) was used for the quantification of spontaneous resistant mutations.

### Vector constructions

Bacterial strains and plasmids (S6 Table), oligonucleotides (S7 Table), and detailed vectors construction (S8 Table) are provided in the corresponding supplementary tables. The pTCV_P_tetO_ vector was used for anhydrotetracycline inducible expression in GBS [66], and the shuttle thermosensitive plasmid pG1 was used for chromosomal deletion, as described previously [67, 68]. Plasmids were constructed by standard restriction and ligation cloning or by Gibson assembly, purified on columns (Qiaprep, Qiagen) and all inserts were sequenced. Plasmids were introduced in GBS by electroporation, except for the Δ*dacA* mutants which were transformed by conjugation with the *E*. *coli* HB101/pRK24 donor strain, as described previously [69], to avoid liquid cultures.

For DRaCALA experiments, *E*. *coli* Bli5 strain was used with the pET-28a (N-terminal His-tag) and pIVEX (N-terminal His-MBP tag) vectors. Similar results were obtained with the two vectors, except for TrkH which is detected by Western only with the His-tag, and OpuCA which give a positive signal by DRaCALA only with the His-MBP tag. For OpuCA, the MBP tag might increase the solubility of the tagged protein, as observed previously with the OpuCA homologue in *S*. *aureus* [27]. For recombinant rDacA, rDacA*, and rBusR purification, *E*. *coli* Bli5 were used with pET28a expression vectors. For *E*. *coli*, antibiotics were used at the following concentrations: ticarcillin, 100 μg/ml; chloramphenicol 30 μg/ml; ampicillin 100 μg/ml; erythromycin, 150 μg/ml; and kanamycin 25 μg/ml.

### GBS deletion and conditional mutants

GBS mutants were constructed with the corresponding thermosensitive pG1 vectors (for *dacA*, *gdpP*, *busA*, *busB*, and *busR* deletion) in three steps, involving: i) selection of transformants at permissive temperature (30°C) with erythromycin; ii) chromosomal integration of the deletion vector at the targeted loci at restrictive temperature (37°C); and iii) decombination and loss of the deletion vector at permissive temperature (30°C) without selective pressure. The final step can give back to a WT allele (defined as the WTb controls) or to deletion of the targeted loci (unmarked deletion). Confirmation of the WTb or deletion genotypes was done by PCR and Sanger sequencing for each mutant.

Attempts to delete *dacA* (*i*.*e*. in-frame deletion of the DacA cytoplasmic domain, codon 106 to 234 of the 283 amino-acids protein) following the standard protocol were unsuccessful, given only WTb colonies at the final step. Therefore, an additional copy of *dacA* was cloned into the conditional pTCV_P_tetO_ expression vector [66] and introduced into the Δ*dacA*::*dacA* intermediate strain (called the integrant) at 30°C with erythromycin and kanamycin (S1 Fig). The final step of losing the integrated vector was repeated in presence of 50 ng/ml aTc and the Δ*dacA* / P_tetO__*dacA* in-frame deletion mutant was obtained at high frequency.

To obtain Δ*dacA* mutants without the P_tetO__*dacA* expression vector, serial cultures in anaerobic condition were done without the selective pressure to maintain the vector (S1 Fig). The P_tetO__*dacA* vector was lost in a WTb background after two serial cultures but all Δ*dacA* / P_tetO__*dacA* retain the vector in the same condition, indicating that a leaky expression of the ectopic *dacA* copy is sufficient to keep a fitness advantage. By testing more than 200 non-pigmented clones after 6 serial cultures on Granada in anaerobic condition, we isolated one Δ*dacA* mutant (Δ*dacA*-1) which has lost the P_tetO__*dacA* vector. An independent Δ*dacA*-2 mutant was obtained from the Δ*dacA*::*dacA* integrant by performing all subsequent steps in anaerobiosis on Granada (S1 Fig). The frequency of Δ*dacA* mutant versus WTb strain was less than 1%, confirming that Δ*dacA* has a fitness disadvantage compared to the WT strain. Finally, the standard protocol was repeated to construct the Δ*dacA*-A, -B and -C mutants except that all steps were done in CDM, resulting in high frequencies of Δ*dacA* mutant.

### Genome sequencing

Genomic DNA was purified from 10 ml of overnight cultures in TH or CDM, except for Δ*dacA*-1 and –2 mutants which were made from colonies on TH plates incubated in anaerobiosis. Bacterial pellets were treated with lysosome (20 mg/ml) and proteinase K before mechanical breaking of the cell by microbeads (FastPrep, MP Biomedicals), and genomic DNA purification (DNeasy Blood, Qiagen) and quantification (Qubit hsDNA, ThermoFisher Scientific). Five micrograms of DNA were used for libraries preparations. The first set of DNA (S1 Table) was treated and sequenced by the Sequencing Core Facilities of Institut Pasteur (Paris, France) with TrueSeq DNA LT kits and single-read sequencing (150 bp) on a MiSeq instrument (Illumina). The second set of DNA (S1 Table) was sheared (Covaris S220 instrument), treated with commercial enzymes and purification kits (Klenow, T4 ligase, T4 polynucleotide kinase, Phusion polymerase from New England Biolabs, and MinElute and QiaQuick columns from Qiagen), ligated to multiplex adapters (NEXTflex, Illumina), and purified (500 bp mean fragment). Paired-end sequencing (2 x 76 bp) was done on a NextSeq 550 apparatus (Illumina). After quality assessments, trimming and de-multiplexing, sequence reads were mapped on the 2.2 Mb reference sequence (RefSeq NC_004368.1) using Geneious software (Biomatters Ltd), resulting in a mean coverage of 131x and 609x for the MySeq and NextSeq instruments, respectively (S1–S4 Tables).

### Differential Radial Capillary Action of Ligand Assay (DRaCALA)

Interaction between c-di-AMP and targeted GBS proteins was tested by DRaCALA [47] on whole *E*. *coli* protein extract. Expression of the candidate GBS protein was done in Bli5 containing pET-28a or pIVEX expression vector (S6 Table). Expression of the tagged-GBS protein was induced with IPTG (1 mM) for 6 hours at 30°C. Bacterial pellet from 1 ml culture is suspended in 100 μl binding buffer (40 mM Tris pH 7.5, 100 mM NaCl, 10 mM MgCl2, 0.5 mg/ml lysozyme, 20 μg/ml DNase), lysed by 3 freeze-thaw cycles, and directly used for DRaCALA and Western blot analysis using anti-His-tag antibodies. For DraCALA, 1 nM ^32^P-labeled c-di-AMP, synthetized as described in reference [22], was added to the whole protein extract, incubated at room temperature for 5 min, and 2.5 μl was spotted onto nitrocellulose membrane. Membranes are revealed with radiographic films (Amersham Hyperfilm ECL, GE Healthcare) and signal intensity quantified with ImageJ (NIH). The c-di-AMP bound fraction was calculated as described [47]. For competition assay 200 μM of cold nucleotides (c-di-AMP, c-di-GMP, cAMP, cGMP, AMP, and ATP; BioLog Life Science Institute, Germany) were added to the protein extract altogether with radiolabelled c-di-AMP.

### c-di-AMP synthesis activity

Recombinant rDacA (amino-acids 96 to 243, deleted from the transmembrane domain) and the mutated rDacA* (with a R_213_K substitution) were expressed as 6xHis N-terminal tagged forms (pET28a vector) in Bli5 *E*. *coli* strain. Cultures were done at 37°C in LB until OD_600_ = 0.7 before protein induction with IPTG (1 mM) for 3 hours. After centrifugation and one cycle of freezing (-20°C), pellets are suspended in 20 ml of buffer (50 mM Na_2_HPO_4_/NaH_2_PO_4_, 300 mM NaCl, pH7.0), and broken by one passage through a French press at 14000 p.s.i. Cell debris were eliminated by centrifugation and the recombinant proteins were purified by chromatography (5 ml TALON crude column, GE Healthcare) with a linear gradient from 0 to 150 mM imidazole in 50 mM Na_2_HPO_4_/NaH_2_PO_4_, 300 mM NaCl, pH7.0, at 5 ml/min for 20 min. Fractions containing the enzyme were pooled and the buffer was exchanged on PD10 column previously equilibrated with 10 mM Bis-Tris, 100 mM NaCl, pH 7.5. Diadenylate cyclase activities were tested at 37°C with 2.5 μM rDacA or rDacA* incubated with 1 mM ATP in 50 mM Tris pH 8.5, 100 mM NaCl and 10 mM MnCl_2_. Formation of c-di-AMP was followed each 14 min by RR-HPLC using a reverse-phase column (Agilent ZORBAX Eclipse XDB-C18, 2.1 x 100 mm, 1.8 μm). Samples were analyzed by RR-HPLC with a flow rate of 0.25 ml/min and a linear gradient of 1–12% acetonitrile (CH3CN) in 20 mM triethylammoniumacetate buffer, pH 7.5. The ATP and c-di-AMP peak areas were used to quantify substrate and product formation.

### c-di-AMP quantification

C-di-AMP quantification in GBS was done by LC-MS/MS (BIOLOG Life Science Institute), following company instructions. Late-exponential GBS cultures (OD_600_ = 0.8) in TH Hepes 50 mM incubated in aerobiosis or anaerobiosis were centrifuged (15 min, 4°C, 2,500 g), and the pellet washed in PBS. Bacteria were suspended in extraction buffer (acetonitrile/methanol/water; 2/2/1), incubated 15 min on ice, heat extracted 10 min at 95°C, and incubated for an additional 15 min on ice. A final mechanical cell lysis step was done with 0.1 mm microbeads with shaking (2 x 30”, FastPrep-24, MP Biomedicals). After centrifugation (10 min, 4°C, 20,000 g), supernatant was transferred into a new tube and the extraction step was repeated twice on cell debris without the heating step. The three supernatants were pooled and store at -20°C overnight to complete protein precipitation. After centrifugation (20 min, 4°C, 20,800 g), the whole extract was evaporated to dryness (Eppendorf concentrator 5301) before quantification by LC-MS/MS. Protein concentration in the bacterial culture was done (Pierce BCA, Thermo Fischer) in parallel to the extraction to normalize c-di-AMP concentration to the total protein content.

### BusR purification and BusR-DNA interaction

Full length recombinant rBusR (amino-acids 1 to 213 tagged with a N-terminal 6xHis) expressed in Bli5 *E*. *coli* strain was purified as rDacA, except that IPTG-induction was done at 20°C overnight, and with an additional purification step by gel filtration (Superdex 10/300 GL, GE Healthcare) after affinity chromatography in a final buffer containing 20 mM Hepes pH 7, 150 mM NaCl. Electrophoretic mobility shift assay (EMSA) was done with a 245 bp PCR fragment (primers pLD1 + pLD2) corresponding to the promoter region of the *busAB* operon (P_*busAB*_). This 5’ region includes the transcription start site and the -10 and -35 boxes, as characterized by whole genome TSS mapping [70]. Primer pLD1 is radiolabelled with T4 polynucleotide kinase (New England Biolabs) and [γ-^32^P]-dATP before PCR reaction. Protein-DNA interaction was done with rBusR, radiolabeled P_*busAB*_ (10^4^ c.p.m), 0.1 μg/μl of Poly(dI-dC) (Pharmacia), and 0.02 μg/μl BSA in binding buffer (25 mM Na_2_HPO_4_/NaH_2_PO_4_ pH 8, 50 mM NaCl, 2 mM MgCl_2_, 1 mM DTT, 10% glycerol) for 20 min at room temperature. Samples were separated onto a 6% polyacrylamide gel for 1 hour at 4°C and analyzed by autoradiography. The same conditions were used for footprinting, with the addition of 62.5 ng/ml DNaseI (Worthington Biochemical) for 30 seconds at room temperature after incubation in the binding buffer. DNaseI treatments were stopped by the addition of 0.4 M sodium acetate, 50 μg ml^−1^ sonicated calf thymus DNA, and 2.5 mM EDTA, before DNA purification by phenol extraction and ethanol precipitation. Purified DNA from each reaction were adjusted to load an equivalent number of radiolabeled product (5 × 10^4^ c.p.m. equivalent) on 6% polyacrylamide/7 M urea sequencing gels. Maxam and Gilbert reactions (A + G) on P_*busAB*_ was carried out as control and gels were analyzed by autoradiography.

### RNA isolation and quantification

Total RNA were extracted from exponentially growing cells (OD_600_ = 0.4) in TH at 37°C (FastRNA ProBlue, MP Biomedicals) and residual DNA removed with the TURBO DNase (Ambion / Thermo Fischer Scientific). RNA were quantified (Nanodrop 2000, Thermo Fischer) before reverse transcription (iScript cDNA synthesis, Bio-Rad). Quantitative PCR (qPCR) was carried out using specific primer pairs (S8 Table) and EvaGreen Universal qPCR Supermix (Bio-Rad) in a CFX96 apparatus (Bio-Rad). Relative quantification of specific gene expression was calculated with the ΔΔCq method, with *gyrA* as the housekeeping reference gene. Results are normalized against the WT strain and each assay was performed in triplicate on three independent cultures.

## Supporting information

S1 Fig Diagram of Δ*dacA* mutants construction. The first step to construct Δ*dacA* mutants is the integration of the thermosensitive deletion vector (pG_Δ*dacA*) at the *dacA* chromosomal locus. The resulting integrant (Δ*dacA*::*dacA*) has a WT copy of *dacA* and an additional in-frame deletion copy. Genomes of independent integrants were sequenced to confirm integration and absence-presence of additional mutations compared to the parental WT strain. (A) The conditional Δ*dacA* / P_tetO__*dacA* mutant was obtained by introducing into the integrant an ectopic vector (pTCV_P_tetO__*dacA*) containing an additional *dacA* copy under the control of the P_tetO_ inducible promoter and by performing the subsequent step in presence of aTc. The Δ*dacA*-1 mutant was obtained in anaerobiosis from the Δ*dacA* / P_tetO__*dacA* mutant by losing the pTCV_P_tetO__*dacA* vector. Δ*dacA*-1 suppressors were selected by plating the Δ*dacA*-1 mutant on TH incubated in aerobiosis. (B) The Δ*dacA*-2 mutant and its isogenic WTb-2 control were obtained on TH incubated in anaerobiosis by losing the pG_Δ*dacA* vector in the integrant. Δ*dacA*-2 suppressors were selected by plating the Δ*dacA*-2 mutant on TH incubated in aerobiosis. (C) The Δ*dacA* and WTb controls (-A to–C) were obtained on minimal media (CDM) in aerobiosis. Erythromycin (Ery_10_) and kanamycin (Km_500_) are used for pG and pTCV_P_tetO_ vectors selection, respectively.
(PDF)

S2 Fig Inactivation of *dacA* is not associated to increase mutation rate or activation of a cryptic diadenylate cyclase. (A) In vitro activity of recombinant rDacA (amino-acids 96 to 243) and of an inactivated form rDacA* (R213K substitution). (B) Frequency of spontaneous mutation. Mutation rates were estimated using a rifampicin resistance assay with overnight cultures platted on TH agar with or without rifampicin (50 μg/ml) incubated in anaerobiosis at 37°C. Mutation rates are the ratios between the number of rifampicin resistant (RifR) colonies and the total number of colonies. (C) Quantification of intracellular c-di-AMP in WT, *ΔdacA* mutants and four suppressors (S30, S34, S35, and S39). Values are mean +/- standard deviation of 3 independent cultures in TH grown in anaerobiosis (blue) or aerobiosis (red) for the WT strain and Δ*gdpP* mutant. Only two independent cultures were tested for the other strains. Quantities of c-di-AMP are normalized against the total protein quantity in the corresponding bacterial extract. N.d: not detected.
(PDF)

S3 Fig Re-expression of WT alleles inhibits growth in Δ*dacA* suppressors. (A) Related to Fig 3H. Conditional expression of a WT copy of mutated genes in 9 Δ*dacA* suppressors (S6, S30, S34, S35, S39, SS43, S44, and S47). Each gene is under the control of a P_tetO_ inducible promoter on a pTCV replicative vector introduced into each suppressor with a mutated allele. Conditional expression was tested by adding aTc (50 ng/ml) in TH on serial dilution of bacterial cultures. Coloured boxes highlight growth inhibition upon expression of a WT allele in aerobiosis and anaerobiosis (red boxes), or aerobiosis only (orange). (B) Control for the conditional expression of each gene in a WT strain under the same condition.
(PDF)

S4 Fig Expression of tagged GBS proteins in *E*. *coli*. Western blots of total protein extract of *E*. *coli* strains expressing tagged GBS proteins with anti-His antibody. For EriC, only the RCK_C domain was successfully expressed.
(PDF)

S5 Fig Inhibitory effect of osmolytes in absence of c-di-AMP synthesis and presence of a functional BusAB transporter. The WT strain and the Δ*dacA*-2 mutant, containing a frameshift mutation in *busB*, were transformed with an empty vector (P_tetO_), or with inducible *dacA* and *busB* complementing vectors (P_tetO__*dacA* and P_tetO__*busB*, respectively), Serial culture dilutions were spotted on TH and CDM media supplemented with 5 mM potassium and 1 mM of osmolytes (glycine betaine, carnitine, or choline), incubated for 24–48 h at 37°C under anaerobiosis or aeobiosis.
(PDF)

S6 Fig *fabT* mutation are selected in CDM medium. (A) Schematic representation of the *fabT* mutations identified by genome sequencing (Illumina) in the three (A, B, C) Δ*dacA*::*dacA* integrants, Δ*dacA* mutants and WTb controls constructed in CDM. (B) Schematic representation of the targeted sequencing of *fabT* (Sanger) in a WT strain grown in TH and in CDM. Two representative chromatograms illustrated the *fabT* WT sequence after grown in TH and the presence of two populations, including one with a *fabT* frameshift, after grow in CDM. (C) Results of *fabT* Sanger sequencing of three independent cultures of the WT strain and of one Δ*dacA*::*dacA* integrant after one and three cultures in TH and CDM at 37°C. Mutations in *fabT* are highlight in red. The relative proportion of strain in the whole population having different mutation is inferred from the relative picks height on Sanger chromatographies.
(PDF)

S1 Table Genome coverage by Illumina sequencing of the ΔdacA mutants (green), the ΔdacA suppressors (pink), and the WT, WTb, and integrants controls (white). (XLSX)

S2 Table Mutations in the WT strain compared to the reference sequence (NC_004368). (XLSX)

S3 Table Mutations in ΔdacA mutants (green), in ΔdacA suppressors (pink), and in integrants and WTb controls (white). (XLSX)

S4 Table Mutations in ΔdacA suppressors organized by genes or functional unit. (XLSX)

S5 Table Chemically defined medium (CDM). (PDF)

S6 Table Bacterial strains and plasmids. (PDF)

S7 Table Primer sequences. (PDF)

S8 Table Plasmid construction. (PDF)
