# Supplementary figures and images for: Cyclic di-AMP regulation of osmotic homeostasis is essential in Group B Streptococcus
Source: PLoS Genet. 2018 Apr 16;14(4):e1007342. doi: 10.1371/journal.pgen.1007342 (PMC5919688; doi:10.1371/journal.pgen.1007342)

**S1 Fig: Diagram of  $\Delta dacA$  mutants construction.**

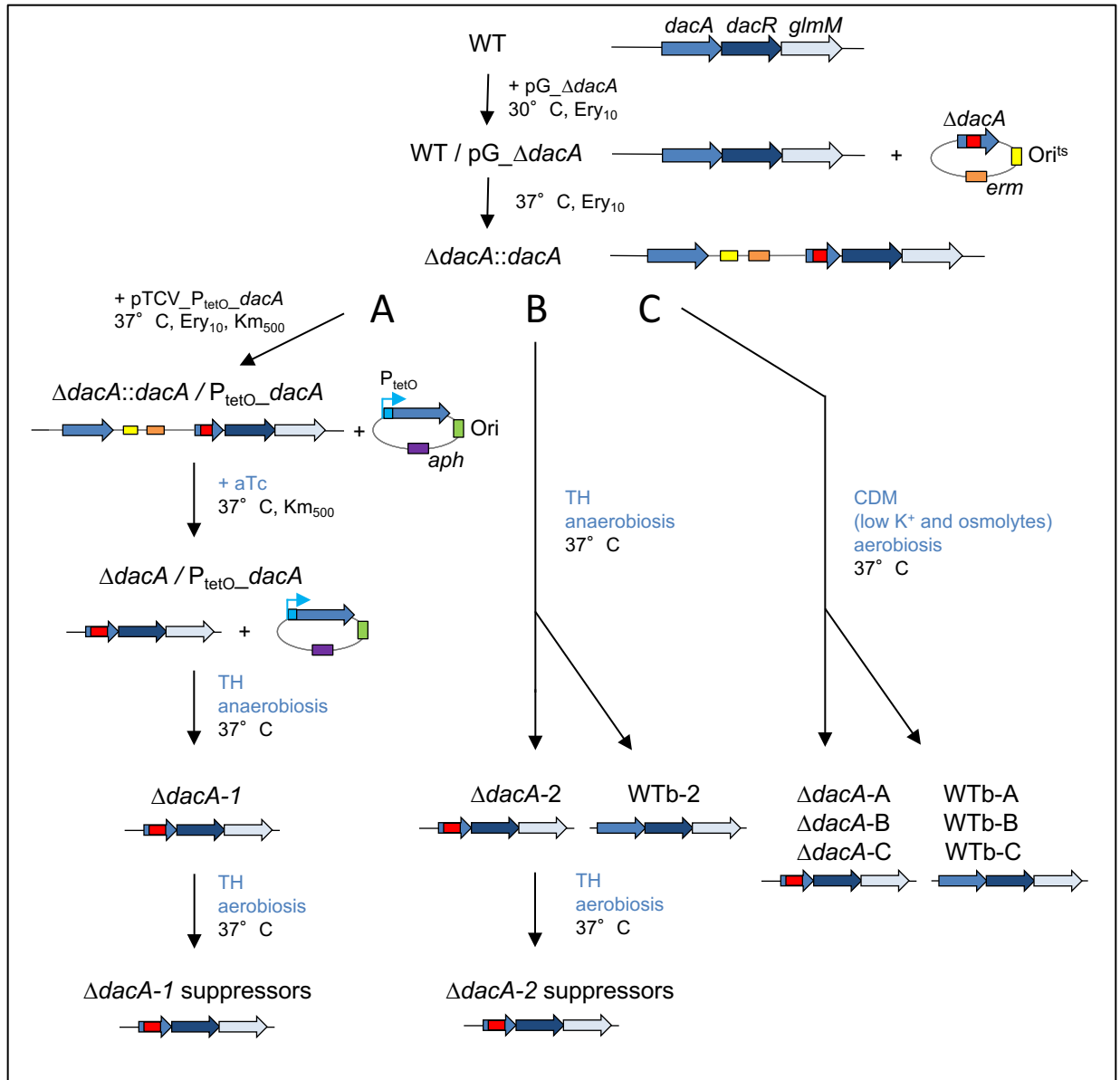

Supplement: S1 Fig — The first step to construct ΔdacA mutants is the integration of the thermosensitive deletion vector (pG_ΔdacA) at the dacA chromosomal locus. The resulting integrant (ΔdacA::dacA) has a WT copy of dacA and an additional in-frame deletion copy. Genomes of independent integrants were sequenced to confirm integration and absence-presence of additional mutations compared to the parental WT strain. (A) The conditional ΔdacA / PtetO_dacA mutant was obtained by introducing into the integrant an ectopic vector (pTCV_PtetO_dacA) containing an additional dacA copy under the control of the PtetO inducible promoter and by performing the subsequent step in presence of aTc. The ΔdacA-1 mutant was obtained in anaerobiosis from the ΔdacA / PtetO_dacA mutant by losing the pTCV_PtetO_dacA vector. ΔdacA-1 suppressors were selected by plating the ΔdacA-1 mutant on TH incubated in aerobiosis. (B) The ΔdacA-2 mutant and its isogenic WTb-2 control were obtained on TH incubated in anaerobiosis by losing the pG_ΔdacA vector in the integrant. ΔdacA-2 suppressors were selected by plating the ΔdacA-2 mutant on TH incubated in aerobiosis. (C) The ΔdacA and WTb controls (-A to–C) were obtained on minimal media (CDM) in aerobiosis. Erythromycin (Ery10) and kanamycin (Km500) are used for pG and pTCV_PtetO vectors selection, respectively. (PDF) [file pgen.1007342.s001.pdf]

S3 Fig. Re-expression of WT alleles inhibits growth in  $\Delta$ acA suppressors.

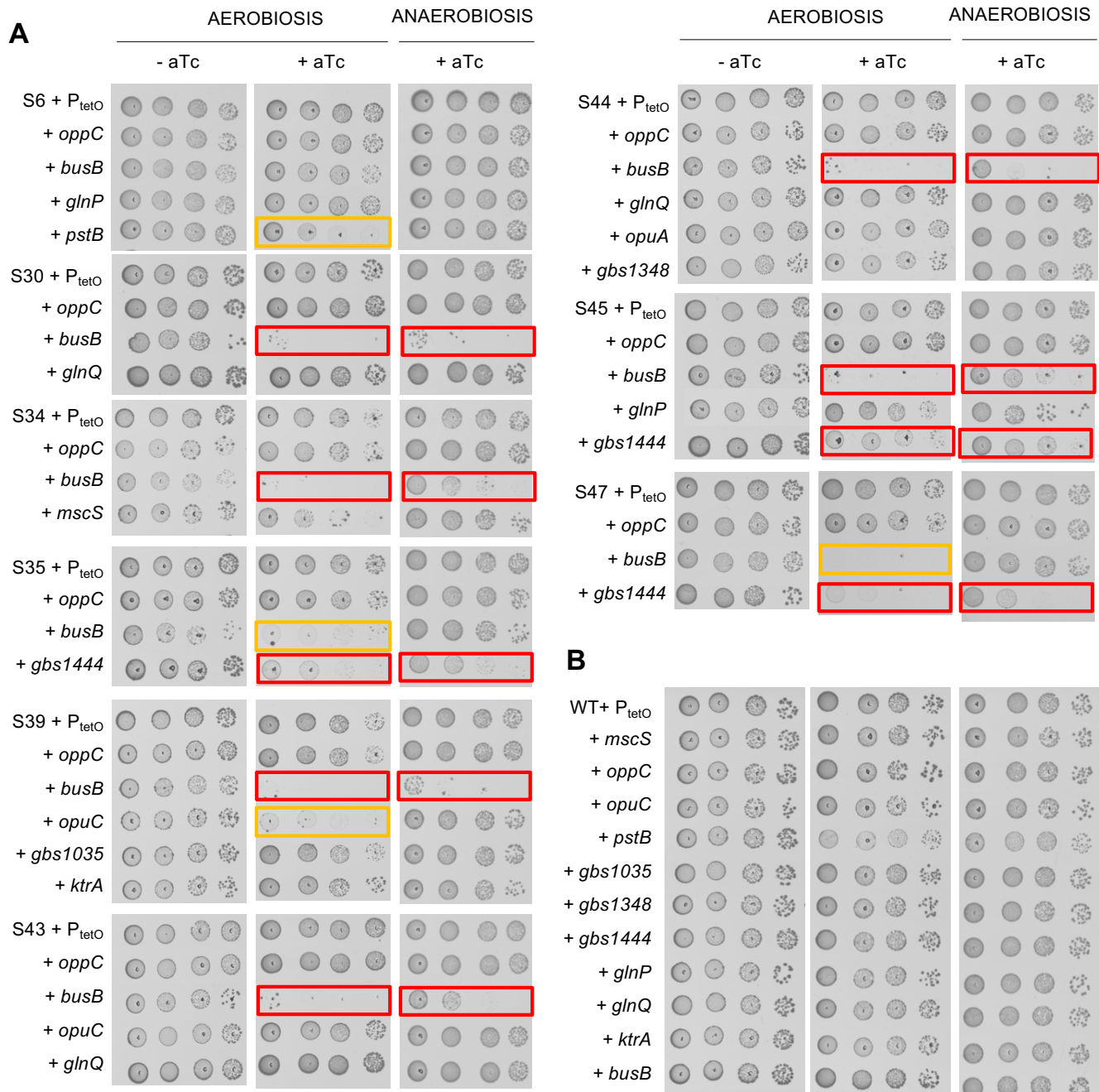

Supplement: S3 Fig — (A) Related to Fig 3H. Conditional expression of a WT copy of mutated genes in 9 ΔdacA suppressors (S6, S30, S34, S35, S39, SS43, S44, and S47). Each gene is under the control of a PtetO inducible promoter on a pTCV replicative vector introduced into each suppressor with a mutated allele. Conditional expression was tested by adding aTc (50 ng/ml) in TH on serial dilution of bacterial cultures. Coloured boxes highlight growth inhibition upon expression of a WT allele in aerobiosis and anaerobiosis (red boxes), or aerobiosis only (orange). (B) Control for the conditional expression of each gene in a WT strain under the same condition. (PDF) [file pgen.1007342.s003.pdf]

**S4 Fig. Expression of tagged GBS proteins in *E. coli*.**

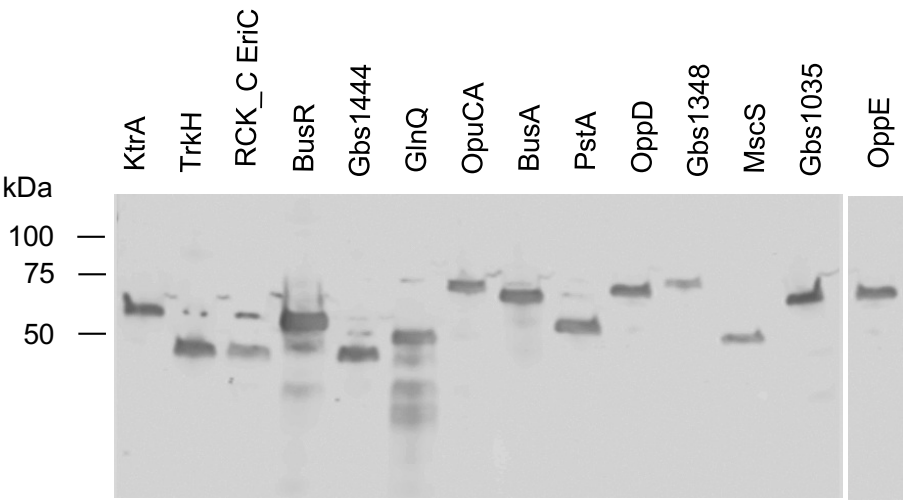

Supplement: S4 Fig — Western blots of total protein extract of E. coli strains expressing tagged GBS proteins with anti-His antibody. For EriC, only the RCK_C domain was successfully expressed. (PDF) [file pgen.1007342.s004.pdf]

**S6 Fig. *fabT* mutation are selected in CDM medium.**

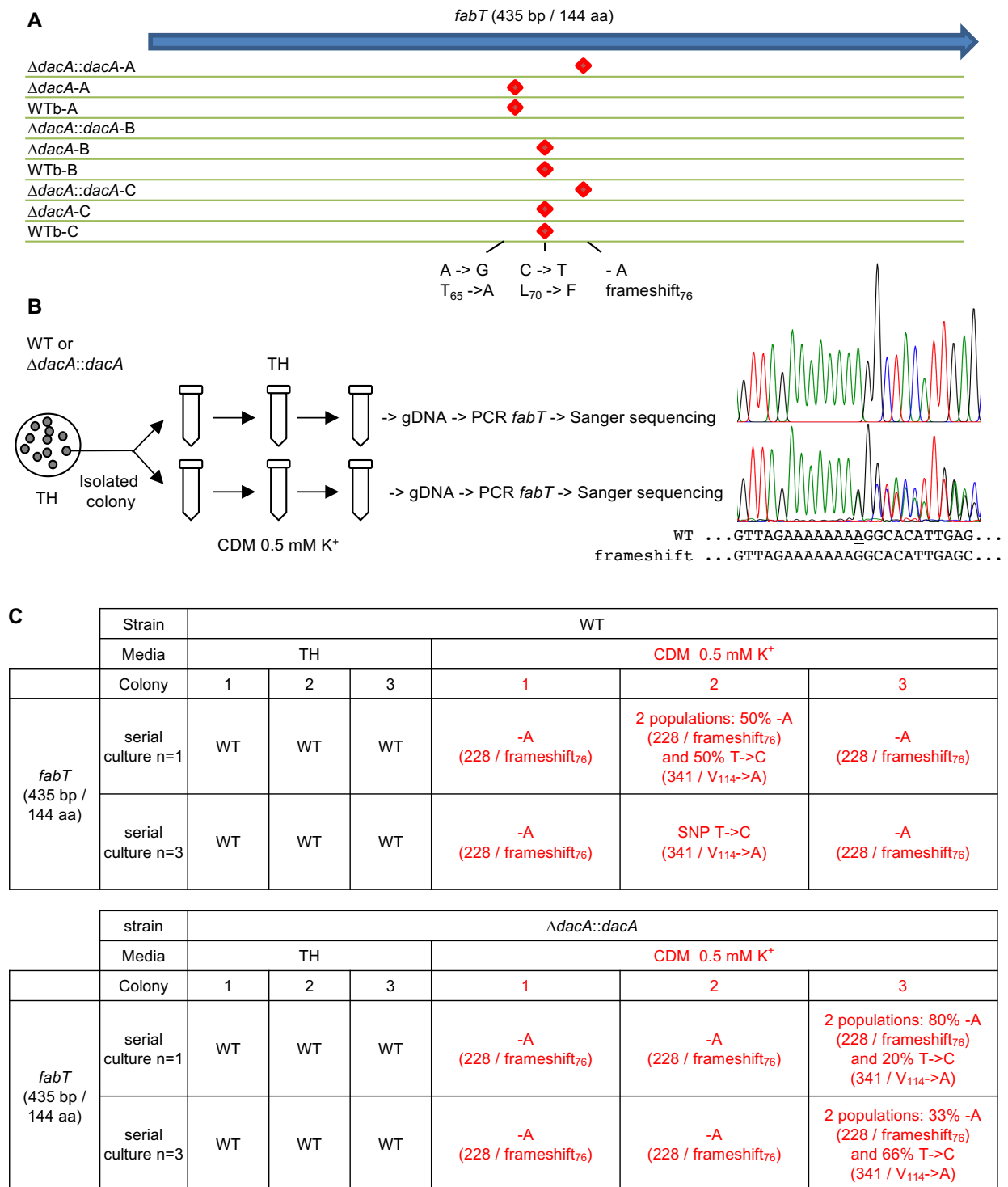

Supplement: S6 Fig — (A) Schematic representation of the fabT mutations identified by genome sequencing (Illumina) in the three (A, B, C) ΔdacA::dacA integrants, ΔdacA mutants and WTb controls constructed in CDM. (B) Schematic representation of the targeted sequencing of fabT (Sanger) in a WT strain grown in TH and in CDM. Two representative chromatograms illustrated the fabT WT sequence after grown in TH and the presence of two populations, including one with a fabT frameshift, after grow in CDM. (C) Results of fabT Sanger sequencing of three independent cultures of the WT strain and of one ΔdacA::dacA integrant after one and three cultures in TH and CDM at 37°C. Mutations in fabT are highlight in red. The relative proportion of strain in the whole population having different mutation is inferred from the relative picks height on Sanger chromatographies. (PDF) [file pgen.1007342.s006.pdf]
