# Supplementary material for: Cyclic di-AMP regulation of osmotic homeostasis is essential in Group B Streptococcus
Source: PLoS Genet. 2018 Apr 16;14(4):e1007342. doi: 10.1371/journal.pgen.1007342 (PMC5919688; doi:10.1371/journal.pgen.1007342)

S2 Fig. Inactivation of dacA is not associated to increase mutation rate or activation of a cryptic diadenylate cyclase.

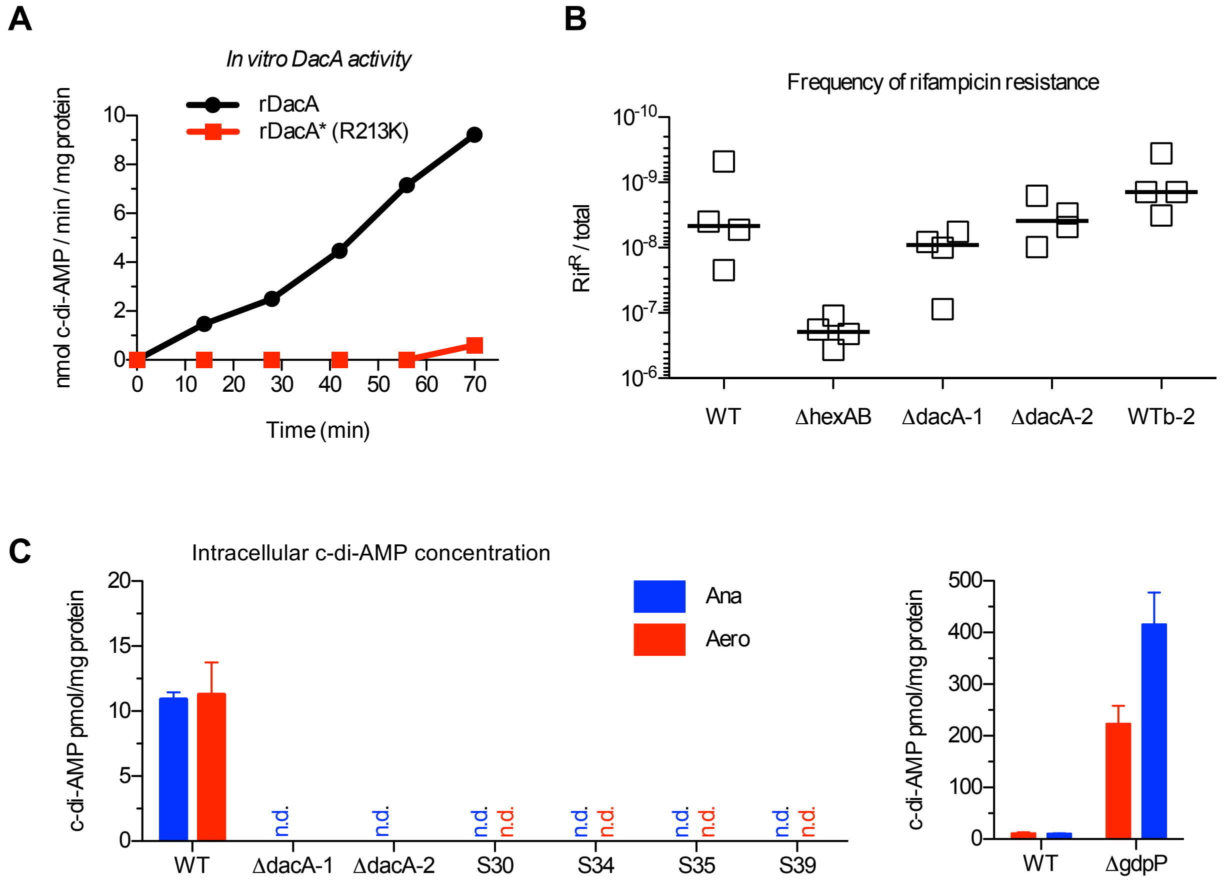

Supplement: S2 Fig — (A) In vitro activity of recombinant rDacA (amino-acids 96 to 243) and of an inactivated form rDacA* (R213K substitution). (B) Frequency of spontaneous mutation. Mutation rates were estimated using a rifampicin resistance assay with overnight cultures platted on TH agar with or without rifampicin (50 μg/ml) incubated in anaerobiosis at 37°C. Mutation rates are the ratios between the number of rifampicin resistant (RifR) colonies and the total number of colonies. (C) Quantification of intracellular c-di-AMP in WT, ΔdacA mutants and four suppressors (S30, S34, S35, and S39). Values are mean +/- standard deviation of 3 independent cultures in TH grown in anaerobiosis (blue) or aerobiosis (red) for the WT strain and ΔgdpP mutant. Only two independent cultures were tested for the other strains. Quantities of c-di-AMP are normalized against the total protein quantity in the corresponding bacterial extract. N.d: not detected. (PDF) [file pgen.1007342.s002.pdf]
