# Supplementary material for: Cyclic di-AMP regulation of osmotic homeostasis is essential in Group B Streptococcus
Source: PLoS Genet. 2018 Apr 16;14(4):e1007342. doi: 10.1371/journal.pgen.1007342 (PMC5919688; doi:10.1371/journal.pgen.1007342)

S5 Fig. Inhibitory effect of osmolytes in absence of c-di-AMP synthesis and presence of a functional BusAB transporter.

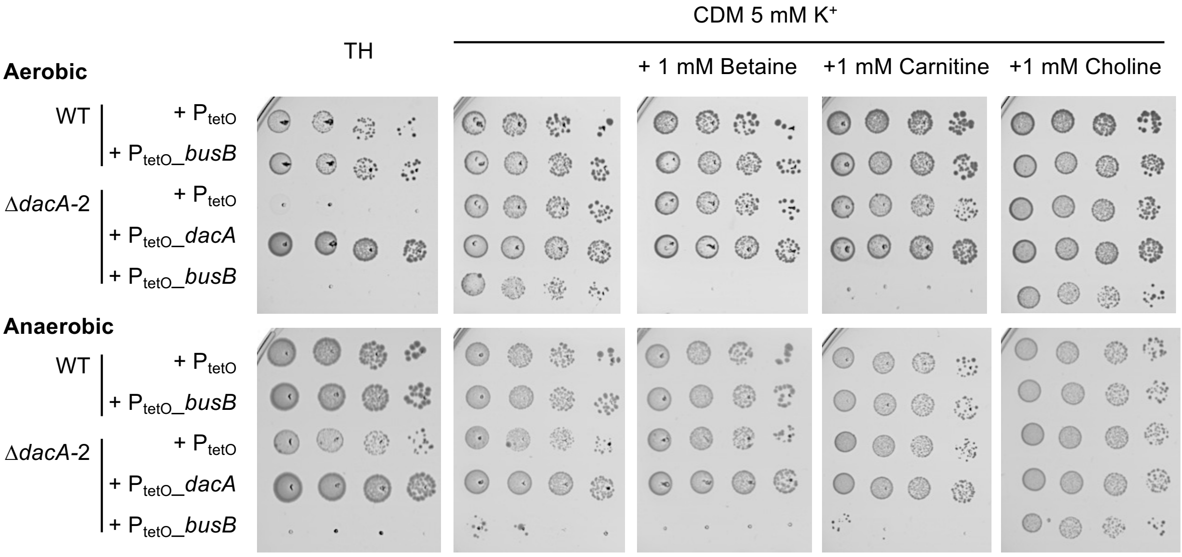

Supplement: S5 Fig — The WT strain and the ΔdacA-2 mutant, containing a frameshift mutation in busB, were transformed with an empty vector (PtetO), or with inducible dacA and busB complementing vectors (PtetO_dacA and PtetO_busB, respectively), Serial culture dilutions were spotted on TH and CDM media supplemented with 5 mM potassium and 1 mM of osmolytes (glycine betaine, carnitine, or choline), incubated for 24–48 h at 37°C under anaerobiosis or aeobiosis. (PDF) [file pgen.1007342.s005.pdf]
