# Supplementary material for: Cyclic di-AMP regulation of osmotic homeostasis is essential in Group B Streptococcus
Source: PLoS Genet. 2018 Apr 16;14(4):e1007342. doi: 10.1371/journal.pgen.1007342 (PMC5919688; doi:10.1371/journal.pgen.1007342)
Supplement: S5 Table — (PDF) [file pgen.1007342.s011.pdf]

Supplementary Table S5: Chemically defined medium (CDM)

Component	Mass (g)	Volume (L)	Solvant	Initiale concentration (g/L)	Volume for 1L of CDM (L)	Final concentration (g/L)
Inorganic Salts						
$CaCl_2$	11.1	0.1	H_2O	111	0.0018	0.1998
NaH_2PO_4	0.5	0.5	H_2O	1	0.1	0.1
$Fe(NO_3)$	0.025	0.25	H_2O	0.1	0.001	0.0001
$MgSO_4$	0.5	0.5	H_2O	1	0.1	0.1
$MnSO_4$	0.25	0.01	H_2O	25	0.001	0.025
$ZnSO_4$	0.1	0.2	H_2O	0.5	0.01	0.005
Amino acids						
L-Alanine	0.4	0.2	H_2O	2	0.05	0.1
L-Asparagine	0.4			2		0.1
L-Aspartic acid	0.6			3		0.15
L-Glutamic acid	0.6			3		0.15
L-Proline	0.4			2		0.1
L-Tryptophan	0.68			3.4		0.17
L-cysteine	1	0.2	0.1N NaOH	5	0.1	0.5
L-Arginine	0.42	0.1	0.2N NaOH	4.2	0.02	0.084
L-Cystine	0.313			3.13		0.0626
Glycine	0.15			1.5		0.03
L-Histidine	0.21			2.1		0.042
L-Isoleucine	0.525			5.25		0.105
L-Leucine	0.525			5.25		0.105
L-Lysine	0.73			7.3		0.146
L-Methionine	0.15			1.5		0.03
L-Phenylalanine	0.33			3.3		0.066
L-Serine	0.21			2.1		0.042
L-Threonine	0.475			4.75		0.095
L-Tyrosine	0.52			5.2		0.104
L-Valine	0.47			4.7		0.094
Vitamins						
Folic acid	0.5	0.01	1N NaOH	50	0.000016	0.0008
Riboflavine	0.1	0.01	0.1N NaOH	10	0.0002	0.002
p-Aminobenzoic acid	0.02	1	H_2O	0.02	0.01	0.0002
Niacinamide	0.1			0.1		0.001
β-NAD	0.25			0.25		0.0025
Pantothenate	0.2			0.2		0.002
Pyridoxal	0.1			0.1		0.001
Pyridoxamine	0.1			0.1		0.001
Thiamine	0.1			0.1		0.001
Vitamin B12	0.01			0.01		0.0001
Biotin	0.02			0.02		0.0002
Bases						
Adenine	0.1	0.1	0.2N NaOH	1	0.01	0.01
Uracil	0.1			1		0.01
Xanthine	0.1			1		0.01
Guanine	0.1			1		0.01
Others						
HEPES	23.8	0.1	H_2O	238	0.05	11.9
Lipoic acid	0.207	0.01	etOH	20.7	0.00012	0.00248
Pyruvate	11	0.1	H_2O	110	0.01	1.1
Glucose	50	0.1	H_2O	500	0.01	5
H_2O					0.525	

*L-Glutamine added just prior to use at 2 mM, final pH adjusted to 7.6

For solid medium, 2X CDM was prepared and mixed v/v with 2X Agar (3% Agar)
