# Supplementary material for: Cyclic di-AMP regulation of osmotic homeostasis is essential in Group B Streptococcus
Source: PLoS Genet. 2018 Apr 16;14(4):e1007342. doi: 10.1371/journal.pgen.1007342 (PMC5919688; doi:10.1371/journal.pgen.1007342)
Supplement: S6 Table — (PDF) [file pgen.1007342.s012.pdf]

Supplementary Table S6: Bacterial strains and plasmids.

Streptococcus agalactiae			
Strain	Relevant genotype	Plasmid	Reference
NEM316	Wild-type, clinical isolate, Serotype III		[1]
NEM3525	New isolate of NEM316	pTCV_P_{tet0}	
NEM3788	WT	pTCV_P_{tet0}	
NEM3353	WT	pTCV_P_{tet0}_*dacA*	
NEM4033	WT	pTCV_P_{tet0}_*mscS*	
NEM4022	WT	pTCV_P_{tet0}_*oppC*	
NEM4735	WT	pTCV_P_{tet0}_*opuCA*	
NEM3952	WT	pTCV_P_{tet0}_*pstA*	
NEM4736	WT	pTCV_P_{tet0}_*gbs1035*	
NEM4737	WT	pTCV_P_{tet0}_*gbs1348*	
NEM4034	WT	pTCV_P_{tet0}_*gbs1444*	
NEM4738	WT	pTCV_P_{tet0}_*glnP*	
NEM4032	WT	pTCV_P_{tet0}_*glnQ*	
NEM4739	WT	pTCV_P_{tet0}_*ktrA*	
NEM4740	WT	pTCV_P_{tet0}_*busB*	
NEM3820	Δ*dacA*	pTCV_P_{tet0}_*dacA*	
NEM3881	Δ*dacA*-1 isolat A		
NEM3884	Δ*dacA*-1 isolat D		
NEM3973	Δ*dacA*-1 S2		
NEM3992	Δ*dacA*-1 S5		
NEM3975	Δ*dacA*-1 S6		
NEM4025	Δ*dacA*-1 S6	pTCV_P_{tet0}_*oppC*	
NEM4741	Δ*dacA*-1 S6	pTCV_P_{tet0}_*busB*	
NEM3964	Δ*dacA*-1 S6	pTCV_P_{tet0}_*glnP*	
NEM3967	Δ*dacA*-1 S6	pTCV_P_{tet0}_*pstA*	
NEM4720	Δ*dacA*-1 S13		
NEM4721	Δ*dacA*-1 S15		
NEM3902	Δ*dacA*::*dacA* clone A		
NEM3903	Δ*dacA*::*dacA* clone B		
NEM3905	Δ*dacA*::*dacA* clone C		
NEM3930	Δ*dacA*-2		
NEM3934	WTb-2		
NEM4790	Δ*dacA*-2	pTCV_P_{tet0}	
NEM4742	Δ*dacA*-2	pTCV_P_{tet0}_*dacA*	
NEM4743	Δ*dacA*-2	pTCV_P_{tet0}_*dacA**	
NEM4744	Δ*dacA*-2	pTCV_P_{tet0}_*oppC*	
NEM4745	Δ*dacA*-2	pTCV_P_{tet0}_*busB*	
NEM3993	Δ*dacA*-2 S30		
NEM4746	Δ*dacA*-2 S30	pTCV_P_{tet0}	
NEM4747	Δ*dacA*-2 S30	pTCV_P_{tet0}_*oppC*	
NEM4748	Δ*dacA*-2 S30	pTCV_P_{tet0}_*busB*	
NEM4028	Δ*dacA*-2 S30	pTCV_P_{tet0}_*glnQ*	
NEM3994	Δ*dacA*-2 S34		
NEM4749	Δ*dacA*-2 S34	pTCV_P_{tet0}	
NEM4750	Δ*dacA*-2 S34	pTCV_P_{tet0}_*oppC*	
NEM4751	Δ*dacA*-2 S34	pTCV_P_{tet0}_*busB*	
NEM4029	Δ*dacA*-2 S34	pTCV_P_{tet0}_*mscS*	
NEM3995	Δ*dacA*-2 S35		
NEM4752	Δ*dacA*-2 S35	pTCV_P_{tet0}	
NEM4753	Δ*dacA*-2 S35	pTCV_P_{tet0}_*oppC*	
NEM4754	Δ*dacA*-2 S35	pTCV_P_{tet0}_*busB*	
NEM4030	Δ*dacA*-2 S35	pTCV_P_{tet0}_*gbs1444*	

NEM3996	Δ*dacA*-2 S39	
NEM4755	Δ*dacA*-2 S39	pTCV_P_{tetO}
NEM4756	Δ*dacA*-2 S39	pTCV_P_{tetO}_*oppC*
NEM4757	Δ*dacA*-2 S39	pTCV_P_{tetO}_*busB*
NEM4758	Δ*dacA*-2 S39	pTCV_P_{tetO}_*opuCA*
NEM4759	Δ*dacA*-2 S39	pTCV_P_{tetO}_*ktrA*
NEM4760	Δ*dacA*-2 S39	pTCV_P_{tetO}_*gbs1035*
NEM3997	Δ*dacA*-2 S43	
NEM4761	Δ*dacA*-2 S43	pTCV_P_{tetO}
NEM4762	Δ*dacA*-2 S43	pTCV_P_{tetO}_*oppC*
NEM4763	Δ*dacA*-2 S43	pTCV_P_{tetO}_*busB*
NEM4764	Δ*dacA*-2 S43	pTCV_P_{tetO}_*opuCA*
NEM4765	Δ*dacA*-2 S43	pTCV_PtetO_*glnQ*
NEM3998	Δ*dacA*-2 S44	
NEM4766	Δ*dacA*-2 S44	pTCV_P_{tetO}
NEM4767	Δ*dacA*-2 S44	pTCV_P_{tetO}_*oppC*
NEM4768	Δ*dacA*-2 S44	pTCV_P_{tetO}_*busB*
NEM4769	Δ*dacA*-2 S44	pTCV_P_{tetO}_*opuCA*
NEM4770	Δ*dacA*-2 S44	pTCV_P_{tetO}_*glnQ*
NEM4771	Δ*dacA*-2 S44	pTCV_P_{tetO}_gbs*1348*
NEM3999	Δ*dacA*-2 S45	
NEM4772	Δ*dacA*-2 S45	pTCV_P_{tetO}
NEM4773	Δ*dacA*-2 S45	pTCV_P_{tetO}_*oppC*
NEM4774	Δ*dacA*-2 S45	pTCV_P_{tetO}_*busB*
NEM4775	Δ*dacA*-2 S45	pTCV_P_{tetO}_*glnP*
NEM4776	Δ*dacA*-2 S45	pTCV_P_{tetO}_*gbs1444*
NEM4000	Δ*dacA*-2 S47	
NEM4777	Δ*dacA*-2 S47	pTCV_P_{tetO}
NEM4778	Δ*dacA*-2 S47	pTCV_P_{tetO}_*oppC*
NEM4779	Δ*dacA*-2 S47	pTCV_P_{tetO}_*busB*
NEM4031	Δ*dacA*-2 S47	pTCV_P_{tetO}_*gbs1444*
NEM4001	Δ*dacA*-2 S48	
NEM4712	Δ*dacA*-A	
NEM4713	WTb-A	
NEM4714	Δ*dacA*-B	
NEM4715	WTb-B	
NEM4718	Δ*dacA*-C	
NEM4719	WTb-C	
NEM4388	Δ*busR*	
NEM4560	Δ*busR*	pTCV_P_{tetO}_*busR*
NEM3691	Δ*gdpP*	
NEM4729	Δ*busA*	
NEM4072	Δ*busB*	
NEM4595	Δ*gdpP* Δ*busR*	
NEM1918	Δ*hexAB*	

Escherichia coli

Strain	Genotype	Reference
TOP10	F- *mcrA Δ(mrr-hsdRMS-mcrBC) φ80lacZΔM15 ΔlacX74 nupG recA1 araD139 Δ(ara-leu)7697 galE15 galK16 rpsL*(Str^R) *endA1* λ^-	Invitrogen
NEB5α	*fhuA2 Δ(argF-lacZ)U169 phoA glnV44 Φ80 Δ(lacZ)M15 gyrA96 recA1 relA1 endA1 thi-1 hsdR17*	New England BioLabs
Bli5	Derived from *E. coli* BL21(DE3), expressed the *lacI* gene on plasmid pDIA17 and the *T7 RNA*	[2]
HB101 pRK24	F^-*hsd-20 recA13 ara-14 proA2 lacY1 galK2 rpsL20* (Str) *xyl-5 mtl-1 supE44*	[3]

DH5∝	F^- *recA1 endA1 gyrA96 thi-1 relA1 Δ(lacIZYA-argF)[φ80ΔlacZΔM15] hsdR17 glnV44 deoR nupG*		Invitrogen
XL1-Blue	*endA1 gyrA96 (nal*^{R}*) thi-1 recA1 relA1 lac glnV44 F'[::Tn10 proAB*$^+$ *lacI*^{q} *Δ(lacZ)M15] hsdR17(*r_K^- m_K^+*)*		Stratagene
**Plasmids published**			
Plasmid	Resistance	Relevant properties	Reference
pRK24	Ampicilin/ Tetracyclin	Tra^+ Mob^+ (IncP)	[4]
pTCV_P_{tetO}	Erythromycin Kanamycin	anhydrotetracycline inducible expression vector, $P_{xyl/tetO}$ promoter	[5]
pG	Erythromycin	*oriR* pUC, *oriR*^{ts} pWV01; MCS pUC18	[6]
pET28a	Kanamycin	Expression vector T7lac promoter, adds N-ter His-tag	Novagen
pIVEX	Ampicilin	Expression vector T7lac promoter, adds N-ter His-MBP-tag	[7]
**Plasmids constructed for this study**			
Plasmid	Relevant properties		
pG_Δ*dacA*	Vector for *dacA* deletion		
pG_Δ*gdpP*	Vector for *gdpP* deletion		
pG_Δ*busR*	Vector for *busR* deletion		
pG_Δ*busA*	Vector for *busA* deletion		
pG_Δ*busB*	Vector for *busB* deletion		
pTCV_P_{tetO}_*dacA*	DacA expression vector, inducible promoter P_{tetO}		
pTCV_P_{tetO}_*dacA**	DacA* (R_{213}K) expression vector, inducible promoter P_{tetO}		
pTCV_P_{tetO}_*oppC*	OppC expression vector, inducible promoter P_{tetO}		
pTCV_P_{tetO}_*busB*	BusB expression vector, inducible promoter P_{tetO}		
pTCV_P_{tetO}_*mscS*	MscS expression vector, inducible promoter P_{tetO}		
pTCV_P_{tetO}_*opuCA*	OpuCA expression vector, inducible promoter P_{tetO}		
pTCV_P_{tetO}_*pstA*	PstA expression vector, inducible promoter P_{tetO}		
pTCV_P_{tetO}_*gbs1035*	Gbs1035 expression vector, inducible promoter P_{tetO}		
pTCV_P_{tetO}_*gbs1348*	Gbs1348 expression vector, inducible promoter P_{tetO}		
pTCV_P_{tetO}_*gbs1444*	Gbs1444 expression vector, inducible promoter P_{tetO}		
pTCV_P_{tetO}_*glnP*	GlnP expression vector, inducible promoter P_{tetO}		
pTCV_P_{tetO}_*glnQ*	GlnQ expression vector, inducible promoter P_{tetO}		
pTCV_P_{tetO}_*ktrA*	KtrA expression vector, inducible promoter P_{tetO}		
pTCV_P_{tetO}_*busR*	BusR expression vector, inducible promoter P_{tetO}		
pIVEX -*ktrA*	Expression vector in *E. coli*, His-MBP tagged protein		
pIVEX -*eriC*	Expression vector in *E. coli*, His-MBP tagged protein		
pIVEX -*busR*	Expression vector in *E. coli*, His-MBP tagged protein		
pIVEX -*gbs1444*	Expression vector in *E. coli*, His-MBP tagged protein		
pIVEX -*glnQ*	Expression vector in *E. coli*, His-MBP tagged protein		
pIVEX -*opuCA*	Expression vector in *E. coli*, His-MBP tagged protein		
pIVEX -*busA*	Expression vector in *E. coli*, His-MBP tagged protein		
pIVEX -*oppE*	Expression vector in *E. coli*, His-MBP tagged protein		
pIVEX -*oppD*	Expression vector in *E. coli*, His-MBP tagged protein		
pIVEX -*gbs1348*	Expression vector in *E. coli*, His-MBP tagged protein		
pIVEX -*mscS*	Expression vector in *E. coli*, His-MBP tagged protein		
pIVEX -*gbs1035*	Expression vector in *E. coli*, His-MBP tagged protein		
pET28a-*trkH*	Expression vector in *E. coli*, His tagged protein		
pET28a-*busR*	Expression vector in *E. coli*, His-tagged protein		

Additional References for Supplementary Table S6

1. Glaser, P., et al., *Genome sequence of Streptococcus agalactiae, a pathogen causing invasive neonatal disease.* Mol Microbiol, 2002. **45**(6): p. 1499-513.
2. Munier, H., et al., *Isolation and characterization of catalytic and calmodulin-binding domains of Bordetella pertussis adenylate cyclase.* Eur J Biochem, 1991. **196**(2): p. 469-74.
3. Boyer, H.W. and D. Roulland-Dussoix, *A complementation analysis of the restriction and modification of DNA in Escherichia coli.* J Mol Biol, 1969. **41**(3): p. 459-72.
4. Thomas, C.M. and C.A. Smith, *Incompatibility group P plasmids: genetics, evolution, and use in genetic manipulation.* Annu Rev Microbiol, 1987. **41**: p. 77-101.
5. Buscetta, M., et al., *FbsC, a Novel Fibrinogen-binding Protein, Promotes Streptococcus agalactiae-Host Cell Interactions.* J Biol Chem, 2014. **289**(30): p. 21003-21015.
6. Danne, C., et al., *Construction of isogenic mutants in Streptococcus gallolyticus based on the development of new mobilizable vectors.* Res Microbiol, 2013. **164**(10): p. 973-8.
7. Roge, J. and J.M. Betton, *Use of pIVEX plasmids for protein overproduction in Escherichia coli.* Microb Cell Fact, 2005. **4**: p. 18.
