# Supplementary material for: Cyclic di-AMP regulation of osmotic homeostasis is essential in Group B Streptococcus
Source: PLoS Genet. 2018 Apr 16;14(4):e1007342. doi: 10.1371/journal.pgen.1007342 (PMC5919688; doi:10.1371/journal.pgen.1007342)
Supplement: S7 Table — (PDF) [file pgen.1007342.s013.pdf]

Supplementary Table S7: Primer sequences.

Oligonucleotide	Sequence (5'-3') *	Features
For plasmids construction		
pAF533	ATGTGAATTCAAGGTAATGAGCAGACTTACCG	EcoRI
pAF534	CAGAAATACCACCAGTTCGCCCAAATTTTTCGAGACCTG	Δ*dacA*
pAF535	CGAAAAATTTGGGCGAACTGGTGGTATTTCTGTCGCTTTG	Δ*dacA*
pAF536	TACTGGATCCTACTACCTTTAGTGGCACTTTGTG	BamHI
pAF316	TCATGAATTCCAGTTTCTGAGACAAATAAACG	EcoRI
pAF317	CATCTTGTAAAAAAATAACTTTCATTAAATAACCTCTTAAGAC	Δ*gdpP*
pAF318	GTCTTAAGAGGTTATTTAATGAAAGTTATTTTTTTACAAGATG	Δ*gdpP*
pAF319	ACTAGGATCCAACAGGTCTTGAGGTTGTACTC	BamHI
pAF752	ATCTGAATTCATGGTGTAAATGTTGAAGTTGGTAC	EcoRI
pAF753	CATCTATATCAAAGACAAAATCCATCCAACCCTCCTTATCCTAATAAAAAC	Δ*busR*
pAF754	GTTTTTATTAGGATAAGGAGGGTTGGATGGATTTTGTCTTTGATATAGATG	Δ*busR*
pAF776	ACATGGATCCTACTTGTCAACTGCAATGGGATTTATG	BamHI
pAF636	AGTAGAATTCGATGGGGAAATCATGCAGATTGGTAC	EcoRI
pAF637	GAGACGTGTTTTTTTTCTATACTATTAAGCTTCTACCGTTTCTTTTGG	Δ*busB*
pAF638	CCAAAAGAAACGGTAGAAGCTTAATAGTATAGAAAAAAAACACGTCTC	Δ*busB*
pAF647	AGTAAGATCTTGGTAATGTCAGAAATTTCTTTTAGC	BglII
pAF871	ACAGCTATGACATGATTACGATGAAGCACAAGCCTTCAGTGATG	Gibson
pAF854	CCAAAATGATTTGTCCTCTCTATCTTATCTCCTACTAAAAATTTCG	Δ*busA*
pAF855	CGAAATTTTTAGTAGGAGATAAGATAGAGAGGACAAATCATTTTGG	Δ*busA*
pAF872	CCTGCAGGTCGACTCTAGAGAGCAAATACAACAGAGGCAAAGAC	Gibson
pAF411	ATCTGGATCCTTCGACAGGCTACCAGTAGAGAG	BamHI
pAF412	ATCACTGCAGATGTCTACTTCCTCCTCAATAGTTG	PstI
pAF604	ATCTGGATCCAAGGAGATGTTATGGCAGATAAAAAC	BamHI
pAF605	AGTACTGCAGCTTATTCATCGCTAGATTTTGGATC	PstI
pAF591	TAGCGTTAACAGATCTGGTAGAGAGGACAAATCATTTTGGA	Gibson
pAF592	CAAGCTTGCATGCCTGCAGTTTTTTTTCTATACTATTTAGTCCACTCTG	Gibson
pAF608	ATCTGGATCCAGGAGTATTACTATGATTACTTTAGAAAAATTTATAG	BamHI
pAF609	TGCTCTGCAGAGGTTTATTATTTATCTC	PstI
pAF606	ATCTAGATCTGTAGAGGAGAATAATATGGGCCAAG	BglII
pAF607	AGTACTGCAGTTATTGATCCTCCGTTTCAGTATCG	PstI
pAF587	ATCTGGATCCATTAAAAGGAGTAGTAATGGCAG	BamHI
pAF588	AGTACTGCAGTCACTATCCAAATCGACCAGAAAC	PstI
pAF730	ACATGGATCCATAGGAAGAGTTATCATGAACGAAATC	BamHI
pAF731	ACATCTGCAGTCTTAGTCTTTTAGAGCATCGAATTTTGC	PstI
pAF732	ACATGGATCCGATAGAGGAGATAAGATGACAAAAATTG	BamHI
pAF733	ACATCTGCAGGCTTGAATTTTTTATCACAGCTCTAAAG	PstI
pAF610	ATCTGGATCCAAGGAGGGCGTCATGTCAAAAGGATATTC	BamHI
pAF611	AGTACTGCAGAAGCTAGGGAACCTAGCTTTTTTAAG	PstI
pAF581	ATCTGGATCCAAAGGAGATTTACATGAAGCACAAG	BamHI
pAF582	AGTACTGCAGTTAATTCTGCCATTATTTAAGCCTC	PstI
pAF612	ATCTGGATCCAAAGAGGCTTAAATAATGGCAG	BamHI
pAF613	AGTACTGCAGTAAAAAAAGATGACACGAAGTCATC	PstI
pAF616	ATCTGGATCCAAAAGGTCTTTTATGAAAACAAAAATTATTG	BamHI
pAF617	AGTACTGCAGCAATAAAAAACTGACCTTAATAGTCAG	PstI
pLD21	ACATGGATCCGATAAGGAGGGTTGGATGGTTTCTGA	BamHI
pLD22	ACATCTGCAGATCCATTCTCTCAAAGCCCCATCCTTA	PstI
DRAC9_Fw	GTGCCGCGCGGCAGCCAGGTTTCTGAACAATCTGAAATTGT	Gibson
DRAC9_Rev	GCTCGAGTGCGGCCGCAAGCTTCAAAGCCCCATCCTTAAGTTAAAA	Gibson
DRAC7_Fw	GTGCCGCGCGGCAGCCAGAGAATAATTGTTGTCGGCGGAGG	Gibson
DRAC7_Rev	GCTCGAGTGCGGCCGCAAGCTCTACCTCGCTAACATATCACTAG	Gibson
pLD24	GGCCGCTTAATTAAACATATGAAAACAAAAATTATTGGTGTACTTG	Gibson
pLD25	GTTACCGGATCCCGGGCTGCAGTTAGTTAAAGTATCCTAAATAGTC	Gibson
pLD78	GGCCGCTTAATTAAACATATGTTGGCTAAAATGACCTTCAATCC	Gibson
pLD79	GTTACCGGATCCCGGGCTGCAGTTACATCAGCTGCTCACGTACTTC	Gibson

pLD28	GGCCGCTTAATTAAACATATGGTTTCTGAACAATCTGAAATTGT	Gibson
pLD29	GTTACCGGATCCCGGGCTGCAGTCAAAGCCCCATCCTTAAGTTAAAA	Gibson
pLD109	GGCCGCTTAATTAAACATATGTCAAAAGGATATTCAAGACAACAG	Gibson
PLD110	GTTACCGGATCCCGGGCTGCAGTTAAGGTGTTGTTGTATTTTC	Gibson
pLD105	GGCCGCTTAATTAAACATATGGCAGAATTAAAAATTGATGTCC	Gibson
pLD106	GTTACCGGATCCCGGGCTGCAGTTAAACGTTTAAAACTTTATTAAGG	Gibson
pLD36	GGCCGCTTAATTAAACATATGGGCCAAGAACCTATCATCGAATATC	Gibson
pLD37	GTTACCGGATCCCGGGCTGCAGTTATTGATCCTCCGTTTCAGTATCG	Gibson
pLD34	GGCCGCTTAATTAAACATATGACAAATATTTTAGAAGTGAAGAAT	Gibson
pLD35	GTTACCGGATCCCGGGCTGCAGTTAAGCTTCTACCGTTTCTTTTGGC	Gibson
pLD103	GGCCGCTTAATTAAACATATGACTGAAAATCGAAAAAAATTAGTTG	Gibson
pLD104	GTTACCGGATCCCGGGCTGCAGTTATAATATTTGTTTTTTATATTC	Gibson
pLD101	GGCCGCTTAATTAAACATATGGAAAAAGAAACTATTTTAAGTG	Gibson
pLD102	GTTACCGGATCCCGGGCTGCAGTTAGGCTTTCCTCCCTTCAATC	Gibson
pLD113	GGCCGCTTAATTAAACATATGACAAAAATTGAAGAAGTGAAGC	Gibson
pLD114	GTTACCGGATCCCGGGCTGCAGTCACAGCTCTAAAGCGGGACTTG	Gibson
pLD107	GGCCGCTTAATTAAACATATGATTACTTTAGAAAAATTTATAGACC	Gibson
pLD108	GTTACCGGATCCCGGGCTGCAGTCAACGAGAGTGGTTATTGGAG	Gibson
pLD111	GGCCGCTTAATTAAACATATGAACGAAATCAAATGCCCTCATTG	Gibson
pLD112	GTTACCGGATCCCGGGCTGCAGTTAGTCTTTTAGAGCATCGAAT	Gibson
rDacA_Fw	ATGAATTCCATATGATTAGAACAGGTCTCGAAAAATTTGGGC	NdeI
rDac_Rev	GGAAGCTTCTACTTCCTCCTCAATAGTTGATTATACCAAGC	HindIII
rDacA*_Fw	CGATTTCAAAAGAATTTGGAACAAAGCACAGAGCAGCGATTGGTCTAC	$R_{213}K$
rDacA*_Rev	GATAGACCAATCGCTGCTCTGTGCTTTGTTCCAAATTCTTTTGAAATCG	$R_{213}K$
BusR_Fw	AGCTGCTAGCATGGTTTCTGAACAATCTGAAATTGTAAC	NheI
BusR_Rev	ATGAAGCTTCAAAGCCCCATCCTTAAGTTAAAATAG	HindIII
For qRT-PCR		
*gyrA*_qF	CCTACTGGTGCCTTGGTGATG	GyrA
*gyrA*_qR	GTGACAACAATACGCTCTTTCCC	GyrA
*busB*_qF	ACAACAAGTGCTTGGTTGCC	BusB
*busB*_qR	TCTCAACGTGAGGACCTAAGTC	BusB
For sequence analysis		
pAF547	GGTTTTACCGTTAGTACCAGTCA	DacA
pAF548	AACTGACATCTTATCGACTGCAT	DacA
pAF320	CGATATTTAAAAAGCGTCAAATG	GdpP
pAF321	CTCACCATAATCAGTTTCTCTGG	GdpP
pAF756	TAGAATCAGTAGACGGACTCTTGG	BusR
pAF757	ACACGTAAATTAGCGTAAGGAGTC	BusR
pAF640	AGACCCATTAATTCGTCGTGAAATG	BusB
pAF641	AATGAGGCTACACAATTAACTGCTG	BusB
pAF857	ATACGCATGGTTACCGTGAAAATG	BusA
pAF858	TTGCTTAGCTGTTGAACCAAATGA	BusA
pLD132	AAGCTTGAATTACAACTGCA	FabT
pLD133	TCAATAAGTTGTTCTGCGAC	FabT
For EMSA		
pLD1	AGTTGTAATATTAATACTAATAAG	P*busAB*
pLD2	AATTTCGTATCTTATTTTTAAGG	P*busAB*
For footprinting		
VlacE	AGTCAAAATAGATATGAACAAATG	P*busAB*
VlacB	GCATTAGTGTATCAACAAGCTGGGG	P*busAB*

* Restriction sites are underlined
