# Supplementary material for: Cyclic di-AMP regulation of osmotic homeostasis is essential in Group B Streptococcus
Source: PLoS Genet. 2018 Apr 16;14(4):e1007342. doi: 10.1371/journal.pgen.1007342 (PMC5919688; doi:10.1371/journal.pgen.1007342)
Supplement: S8 Table — (PDF) [file pgen.1007342.s014.pdf]

Supplementary Table S8: Plasmid construction.

1st PCRs	Matrix	2nd PCR	Digestion	Vector	Analysis
Construction of pGΩΔ*dacA*					
a) pAF533 +	gDNA NEM316	pAF533 +	EcoRI	pG1	pAF547
pAF534		pAF536	BamHI		pAF548
b) pAF535 +	gDNA NEM316				
pAF536					
Construction of pGΩΔ*gdpP*					
a)pAF316 +	gDNA NEM316	pAF316 +	EcoRI	pG1	pAF320
pAF317		pAF319	BamHI		pAF321
b) pAF318 +	gDNA NEM316				
pAF319					
Construction of pGΩΔ*busB*					
a) pAF636 +	gDNA NEM316	pAF636 +	EcoRI	pG1	pAF640
pAF637		pAF647	BglII		pAF641
b) pAF638 +	gDNA NEM316				
pAF647					
Construction of pGΩΔ*busA*					
a) pAF871 +	gDNA NEM316	pAF871 +	Gibson	pG1	pAF857
pAF854		pAF872	assembly		pAF858
b) pAF855 +	gDNA NEM316				
pAF872					
Construction of pGΩΔ*busR*					
a) pAF752 +	gDNA NEM316	pAF752 +	EcoRI	pG1	pAF756
pAF753		pAF776	BamHI		pAF757
b) pAF754 +	gDNA NEM316				
pAF776					
Construction of pTCV_P_{tetO}_*dacA*					
pAF411 +	gDNA NEM316		BamHI	pTCV_P_{tetO}	
pAF412			PstI		
Construction of pTCV_P_{tetO}_*dacA**					
a) pAF411+	gDNA NEM316	pAF411 +	BamHI	pTCV_P_{tetO}	
rDacA*_Fw		pAF412	PstI		
b) pAF412 +					
rDacA*_Rev					
Construction of pTCV_P_{tetO}_*oppC*					
pAF604 +	gDNA NEM316		BamHI	pTCV_P_{tetO}	
pAF605			PstI		
Construction of pTCV_P_{tetO}_*busB*					
pAF591	gDNA NEM316		Gibson	pTCV_P_{tetO}	
pAF592			assembly		
Construction of pTCV_P_{tetO}_*mscS*					
pAF608 +	gDNA NEM316		BamHI	pTCV_P_{tetO}	
pAF609			PstI		
Construction of pTCV_P_{tetO}_*opuCA*					
pAF606+	gDNA NEM316		BglII	pTCV_P_{tetO}	
pAF607			PstI		
Construction of pTCV_P_{tetO}_*pstB*					
pAF587+	gDNA NEM316		BglII	pTCV_P_{tetO}	
pAF588			PstI		
Construction of pTCV_P_{tetO}_*gbs1035*					
pAF730+	gDNA NEM316		BglII	pTCV_P_{tetO}	
pAF731			PstI		
Construction of pTCV_P_{tetO}_*gbs1348*					
pAF732+	gDNA NEM316		BglII	pTCV_P_{tetO}	
pAF733			PstI		
Construction of pTCV_P_{tetO}_*gbs1444*					
pAF610+	gDNA NEM316		BamHI	pTCV_P_{tetO}	

pAF611		PstI	
Construction of pTCV_P_{tetO}_*glnP*			
pAF581+	gDNA NEM316	BamHI	pTCV_P_{tetO}
pAF582		PstI	
Construction of pTCV_P_{tetO}_*glnQ*			
pAF612+	gDNA NEM316	BamHI	pTCV_P_{tetO}
pAF613		PstI	
Construction of pTCV_P_{tetO}_*ktrA*			
pAF616+	gDNA NEM316	BamHI	pTCV_P_{tetO}
pAF617		PstI	
Construction of pTCV_P_{tetO}_*busR*			
pLD21 +	gDNA NEM316	BamHI	pTCV_P_{tetO}
pLD22		PstI	
Construction of pIVEX -*ktrA*			
pLD24 +	gDNA NEM316	Gibson	pIVEX
pLD25		assembly	
Construction of pIVEX –*eriC*_(RCK_C)			
pL78 +	gDNA NEM316	Gibson	pIVEX
pLD79		assembly	
Construction of pIVEX -*busR*			
pLD28 +	gDNA NEM316	Gibson	pIVEX
pLD29		assembly	
Construction of pIVEX -*gbs1444*			
pLD109 +	gDNA NEM316	Gibson	pIVEX
pLD110		assembly	
Construction of pIVEX -*glnQ*			
pLD105 +	gDNA NEM316	Gibson	pIVEX
pLD106		assembly	
Construction of pIVEX -*opuCA*			
pLD36 +	gDNA NEM316	Gibson	pIVEX
pLD37		assembly	
Construction of pIVEX -*busA*			
pLD34 +	gDNA NEM316	Gibson	pIVEX
pLD35		assembly	
Construction of pIVEX -*oppE*			
pLD103 +	gDNA NEM316	Gibson	pIVEX
pLD104		assembly	
Construction of pIVEX -*oppD*			
pLD101 +	gDNA NEM316	Gibson	pIVEX
pLD102		assembly	
Construction of pIVEX -*gbs1348*			
pLD113 +	gDNA NEM316	Gibson	pIVEX
pLD114		assembly	
Construction of pIVEX -*mscS*			
pLD107 +	gDNA NEM316	Gibson	pIVEX
pLD108		assembly	
Construction of pIVEX -*gbs1035*			
pLD111 +	gDNA NEM316	Gibson	pIVEX
pLD112		assembly	
Construction of pET28a-*trkH*			
DRAC7_Fw +	gDNA NEM316	Gibson	pET28a
DRAC7_Rev		assembly	
Construction of pET28a-*busR*			
DRAC9_Fw +	gDNA NEM316	Gibson	pET28a
DRAC9_Rev		assembly	
Construction of pET28a-r*dacA*			
rDacA_Fw +	gDNA NEM316	NdeI	pET28a
rDacA_Rev		HindIII	

Construction of pET28a-r*dacA**			
rDacA*_Fw + rDacA*_Rev	pET28a-r*dacA*	DpnI	pET28a
